# Executive Functions and Impulsivity as Transdiagnostic Correlates of Psychopathology in Childhood: A Behavioral Genetic Analysis

**DOI:** 10.3389/fnhum.2022.863235

**Published:** 2022-03-25

**Authors:** Samantha M. Freis, Claire L. Morrison, Harry R. Smolker, Marie T. Banich, Roselinde H. Kaiser, John K. Hewitt, Naomi P. Friedman

**Affiliations:** ^1^Institute for Behavioral Genetics, University of Colorado, Boulder, Boulder, CO, United States; ^2^Department of Psychology and Neuroscience, University of Colorado, Boulder, Boulder, CO, United States; ^3^Institute of Cognitive Science, University of Colorado, Boulder, Boulder, CO, United States; ^4^Renee Crown Wellness Institute, University of Colorado, Boulder, Boulder, CO, United States

**Keywords:** self-regulation, cognitive control, executive control, behavior problems, heritability

## Abstract

Executive functions (EFs) and impulsivity are dimensions of self-regulation that are both related to psychopathology. However, self-report measures of impulsivity and laboratory EF tasks typically display small correlations, and existing research indicates that impulsivity and EFs may tap separate aspects of self-regulation that independently statistically predict psychopathology in adulthood. However, relationships between EFs, impulsivity, and psychopathology may be different in childhood compared to adulthood. Here, we examine whether these patterns hold in the baseline assessment of the Adolescent Brain and Cognitive Development (ABCD) sample, a national sample of over 11,000 children (including 749 twin pairs) ages 9–10 years. We examine the phenotypic and genetic relationships among latent variables for different components of EFs and multiple facets of impulsivity. Additionally, we assess how EFs and impulsivity relate to composite measures and latent variables of psychopathology derived from parent report. EFs were weakly correlated with impulsivity, and the strength varied by impulsivity facet, emphasizing their separability. We did not identify significant genetic and environmental correlations between EFs and impulsivity. Moreover, controlling for their small relationships with each other, both EFs and some facets of impulsivity statistically predicted an Externalizing factor, attention problems, and social problems, and twin analyses suggested these relationships were genetic in origin. These findings indicate that EFs and impulsivity represent phenotypically and genetically separable aspects of self-regulation that are both transdiagnostic correlates of psychopathology in childhood.

## Introduction

Multiple symptoms of psychopathology may arise due to failure to regulate goal-directed behavior in the face of distraction or opposing urges (Snyder et al., 2015). For example, substance use disorder involves difficulty maintaining abstinence goals in the face of substance-related cues (Kim-Spoon et al., 2017). This ability to control goal-directed behavior has been a topic of extensive research in multiple traditions of psychology. For example, cognitive psychology researchers focus on executive functions (EFs), the “high-level cognitive processes that, through their influence on lower-level processes, enable individuals to regulate their thoughts and actions during goal-directed behavior” (Friedman and Miyake, 2017). Meanwhile, personality psychology researchers focus on forms of behavioral control, like impulsivity, the “non-reflective selection of the stimulus-evoked response,” or the “non-reflective selection or preference for the immediately rewarding response” (Nigg, 2017, p. 370). Though conceptually similar and often related to the broader construct of self-regulation (Nigg, 2017), these cognitive and behavioral aspects of control are typically measured via different methods and only weakly correlate with each other (*r*s = –0.14–0.25) (Duckworth and Kern, 2011; Harden et al., 2017; Malanchini et al., 2018; Friedman et al., 2020). Yet both aspects of control correlate with a broad range of psychopathology and psychiatric symptoms (Moffitt et al., 2011; Nigg, 2017; Strauman, 2017). Although work with adults suggests EFs and impulsivity may be distinct and independently associated with psychopathology, it is unclear if these patterns hold in childhood. In this study, we examined phenotypic, genetic, and environmental correlations between EFs and impulsivity, and their relationships to psychopathology in the large and diverse ABCD Study. We tested the hypothesis that these control-related constructs are genetically separable and independently predict multiple aspects of psychopathology in childhood.

## Transdiagnostic Models of Psychopathology

Akin to the way that factor analyses were used to identify factors underlying intelligence (Spearman, 1904), recent work has taken a similar approach to identify the factors underlying psychopathology. Multiple types of factor models can be used to examine questions of transdiagnostic risk and relate psychopathology to other constructs, including correlated factors, second-order hierarchical, and bifactor models (Brunner et al., 2012; Kline, 2016; Bornovalova et al., 2020). Bifactor models have become popular (Caspi et al., 2014; Snyder et al., 2015; Lahey et al., 2017; Harden et al., 2020) because they capture common variance across psychopathology measures in a single factor, called the *p*-factor (Caspi et al., 2014). In a bifactor model of transdiagnostic risk across psychopathology domains, a *p*-factor is specified to predict all symptoms, so it captures common liability across psychopathology symptoms. In addition, specific orthogonal factors are specified to predict additional variance (i.e., not captured by the *p*-factor) in symptoms within discrete categories. For example, Internalizing-specific and Externalizing-specific factors are often specified to capture variance unique to internalizing psychopathology (e.g., depression and anxiety) or externalizing psychopathology (e.g., substance use disorders and antisocial personality disorder), respectively. Such models have been used to test the hypothesis that EFs are transdiagnostically related to psychopathology by examining whether EFs are significantly associated with the *p*-factor (Hatoum et al., 2018; Snyder et al., 2019; Harden et al., 2020).

However, some researchers have critiqued bifactor models of psychopathology, arguing that the factors may not have clear interpretations or biological substrates (van Bork et al., 2017; Watts et al., 2019; Grotzinger et al., 2020). Correlated factors models of psychopathology can be simpler to interpret compared to bifactor models (Bornovalova et al., 2020). In a correlated factors model, different types of psychopathology are predicted by different factors (e.g., Internalizing and Externalizing Psychopathology), and their shared variance is represented in the correlations between the factors. Although these models do not capture common variance in a single *p*-factor, they can still be used to test for transdiagnostic associations: If EFs relate to multiple factors in a correlated factors model, a researcher could conclude that they are transdiagnostic correlates of psychopathology. For example, Hatoum et al. (2018) found that in males, general EF was associated with *both* the Internalizing and Externalizing Psychopathology factors in a correlated factors model. Consistent with this transdiagnostic association in the correlated factors model, they found that in males general EF was associated with the *p*-factor in a bifactor model of the same data. These consistent associations across modeling strategies suggest that general EF is a transdiagnostic correlate of psychopathology.

The structure of psychopathology has been examined across development (Lahey et al., 2017). In a longitudinal sample spanning childhood and adolescence, Hatoum et al. (2018) estimated correlated factors and bifactor *p*-factor growth models for parent and teacher ratings of internalizing and externalizing psychopathology symptoms. Bifactor *p*-factor models have been examined for both self and parent-reported psychopathology symptoms in childhood and adolescence (Harden et al., 2020). Overall, *p*-factor models of psychopathology have been found to be applicable in childhood and adolescence, show developmental stability in adolescence (Snyder et al., 2017), and identify similar factors (Internalizing, Externalizing, and the *p*-factor) as models examined in adults (Lahey et al., 2017).

Researchers who have compared alternative factor models of psychopathology applied to the baseline ABCD study data concluded that the practical differences between these modeling strategies might be negligible (Clark et al., 2021). In the current study, we employed multiple methods of modeling psychopathology including using composite scores, a correlated factors model, and a bifactor model. We present the results from the correlated factors model in the main text, and the results from models with the composites or bifactor model in the Supplementary Materials. Results across modeling strategies were largely consistent, and we discuss possible interpretations of the results of both modeling strategies.

## Executive Functions and Psychopathology

Executive functions impairments are observed in diverse forms of psychopathology (Snyder et al., 2015; Nigg, 2017). However, most clinical research has relied on examining associations between psychopathology and performance on individual neuropsychology tasks and has not differentiated amongst separable factors of EFs (Snyder et al., 2015). The unity/diversity model of EFs (Friedman and Miyake, 2017) decomposes variance in EF tasks designed to tap response inhibition, working memory updating, and mental set shifting into a factor predicting all EF tasks (Common EF; i.e., unity). Once this common variance is accounted for, two specific factors (Shifting-Specific and Updating-Specific; i.e., diversity) are also needed to account for remaining covariance (i.e., not explained by Common EF) among shifting and updating tasks, respectively. In these studies, there is no inhibition-specific variance left over above and beyond the influence of Common EF, indicating that Common EF may be particularly important for performance on inhibition tasks (Friedman and Miyake, 2017). Individual differences in Common EF are proposed to reflect differences in the ability to form and maintain goals and use those goals to bias ongoing processing, often in the face of distraction (Friedman and Miyake, 2017). The unity/diversity model is well replicated (Miyake and Friedman, 2012; Friedman and Miyake, 2017) and observed in samples as young as middle childhood (Engelhardt et al., 2015; Freis et al., 2022).

Studies incorporating latent variable models of EFs and behavioral and clinical outcomes typically find that the Common EF factor is more relevant to the studied outcome than specific EFs such as the Updating-specific factor (Friedman and Miyake, 2017; Hatoum et al., 2018; Friedman et al., 2020). For example, in the Hatoum et al. (2018) study mentioned earlier, it was the Common EF factor, but not the Updating-specific or Shifting-specific factors, that was associated with Internalizing and Externalizing factors in a correlated factors model and a *p*-factor in a bifactor model. Across studies examining youth, adolescents, or young adults, Common EF is typically negatively related to the *p*-factor, indicating that lower Common EF may be a transdiagnostic correlate of psychopathology (Hatoum et al., 2018; Snyder et al., 2019; Harden et al., 2020). Furthermore, behavioral genetic literature suggests that lower Common EF and higher impulsivity may confer genetic risk for psychopathology (Friedman et al., 2020; Harden et al., 2020).

## Impulsivity and Psychopathology

Impulsivity reflects a breakdown in control, leading to difficulties in reflecting before acting, resisting distraction, and remaining focused on a goal (Nigg, 2017; Watts et al., 2020). Impulsivity is a multifaceted construct (Whiteside and Lynam, 2001). Four commonly studied dimensions of impulsivity include (lack of) premeditation or planning, the “tendency to delay action in favor of careful thinking and planning”; sensation seeking, the “tendency to seek excitement and adventure”; (lack of) perseverance, reflecting “one’s ability to remain with a task until completion and avoid boredom”; and urgency, the “tendency to commit rash or regrettable actions as a result of intense negative affect”; (Whiteside and Lynam, 2001, p. 677). A distinction between positive and negative urgency was added later, leading to five dimensions of impulsivity (Lynam et al., 2006). Consistent with results of studies of adults, impulsivity dimensions are separable in childhood (Watts et al., 2020). These impulsivity dimensions are related to different aspects of personality, behavior problems and psychopathology, and neurocognitive functioning in childhood and adulthood (DeYoung and Rueter, 2010; Friedman et al., 2020; Watts et al., 2020). Additionally, in adulthood, both twin and molecular genetic studies have demonstrated that the different facets of impulsivity are genetically separable (Friedman et al., 2020; Gustavson et al., 2020).

Different impulsivity dimensions may assess different components of the dual systems model of adolescent risk-taking. The dual systems model proposes that risk-taking behavior is due to two complementary systems: an early-maturing affective system that increases one’s affinity for reward, novelty, and sensation seeking and a more slowly developing cognitive control system (Steinberg, 2010; Shulman et al., 2016). It may be that risk-taking peaks in adolescence due to this discordance in developing the bottom-up processing of rewards and the top-down cognitive control system (Shulman et al., 2016). More broadly, self-regulation has also been proposed to involve top-down and bottom-up processes mutually influencing each other (Nigg, 2017). With respect to impulsivity dimensions, urgency and lack of planning may be more related to the top-down regulation of emotion and behavior, while sensation seeking may be more associated with the bottom-up processing of rewards (Smith and Cyders, 2016; Harden et al., 2017).

High impulsivity or deficits in impulse control are frequently posited as risk factors for or correlates of psychopathology in childhood (Beauchaine and Neuhaus, 2008), adulthood (Berg et al., 2015), and across development (Nigg, 2017). However, some components of impulsivity may be transdiagnostic risk factors for psychopathology, while others may be related to specific psychopathology symptoms. For example, high urgency is consistently associated with several psychiatric outcomes and increased psychopathology symptoms in adults (Fischer et al., 2008; Berg et al., 2015; Friedman et al., 2020). Moreover, urgency is consistently genetically correlated with psychopathology in adulthood (Friedman et al., 2020; Gustavson et al., 2020). On the other hand, sensation seeking may be more relevant to specific psychopathology symptoms like externalizing behaviors and substance use in adults (Berg et al., 2015; Friedman et al., 2020). In childhood, trait impulsivity may confer risk for ADHD and externalizing behaviors like conduct problems. Moreover, impulsivity coupled with emotion dysregulation, an expression of emotion that interferes with goal-directed behavior, may confer risk for the development of externalizing spectrum disorders (Beauchaine, 2015).

## Impulsivity and Executive Functions

As reviewed in earlier sections, both EFs (usually based on laboratory tasks) and some aspects of impulsivity (based on questionnaire ratings) are transdiagnostically associated with psychopathology. It is tempting to interpret such results as evidence that EFs and impulsivity, though measured differently, tap some common variance in control that is related to psychopathology. Surprisingly, however, rating and task measures of control typically display small correlations (Duckworth and Kern, 2011; Harden et al., 2017; Friedman et al., 2020).

There are several possible interpretations of these low correlations between task-based and rating-based measures of control, including methodological and conceptual issues (Barkley and Fischer, 2011; Nęcka et al., 2012; Suchy et al., 2017). Reliability issues are a particular challenge for EF tasks (Enkavi et al., 2019). However, low reliability may not entirely explain low correlations between EF tasks and ratings, as latent variables of EFs and self-reported control are still only modestly correlated (Snyder et al., 2020). Latent variables remove random measurement error, so if low reliability of EF tasks is the main issue underlying low correlations between tasks and ratings, employing latent variables should lead to higher correlations.

Low validity has also been proposed as an underlying issue for both EF tasks and self-reports. Some researchers argue that EF tasks have low ecological validity because they are administered in extremely controlled laboratory settings whereas ratings assess more “real-world” control abilities, while others suggest that self-reports have low validity because individuals have poor awareness of their own self-regulatory abilities (Barkley and Fischer, 2011; Nęcka et al., 2012; Suchy et al., 2017). However, task and rating-based measures of EFs and impulsivity both independently relate to relevant behavioral and academic outcomes like Externalizing psychopathology and reading ability (Malanchini et al., 2018; Friedman et al., 2020).

Another piece of evidence that the separability of task and rating-based measures of EFs and impulsivity may reflect more than method variance comes from behavioral genetic analyses. Behavioral genetic models, further described in the statistical procedures section, leverage different degrees of genetic relatedness from identical and fraternal twins to estimate the proportion of variance in an outcome that is attributable to genetic and environmental influences. Twin studies provide aggregate estimates of genetic and environmental influences on a trait (Friedman et al., 2021). Additionally, behavioral genetic models can be used to estimate genetic and environmental correlations between multiple outcomes. Specifically, control-related constructs assessed via different methods also display weak to moderate genetic correlations in adulthood and childhood (Malanchini et al., 2018; Friedman et al., 2020). Because genetic correlations focus on the variance in each measure that is both reliable and related to genetic differences, such patterns suggest that this separability remains after accounting for methodological issues like random measurement error and variance from environmental factors that may be different across measurement method.

Taken together, the evidence reviewed above suggests that the small correlations between EFs and impulsivity are unlikely to be explained by reliability or validity problems in one or both types of measures. An alternative explanation is that impulsivity and EFs tap distinct aspects of control that could be independently related to outcomes like psychopathology (Sharma et al., 2014; Friedman et al., 2020). For example, EF tasks administered in laboratory settings may assess optimal performance in goal-maintenance and top-down biasing, while self-regulation and impulsivity self-report questionnaires may assess more day-to-day control abilities (Samyn et al., 2015; Friedman and Gustavson, 2022). Additionally, some researchers have proposed that self-report measures assess emotional and motivational mechanisms, while behavioral tasks assess cognitive mechanisms (Sharma et al., 2014). If it were the case that EFs and impulsivity assess different aspects of control, they should not predict the same variance in psychopathology. Examining this question requires including these different aspects of control in the same model; however, studies integrating both task-based and ratings-based measures of cognitive and behavioral control are relatively rare.

Existing research has simultaneously examined relationships between EFs, impulsivity, and psychopathology in adulthood (Friedman et al., 2020) and relationships between EFs, impulsivity, and academic outcomes in childhood (Malanchini et al., 2018). However, to our knowledge, no study has jointly examined phenotypic, genetic, and environmental relationships between multiple aspects of control and psychopathology in childhood. In a childhood twin sample, Malanchini et al. (2018) found positive but small phenotypic correlations between a task-based Common EF factor and latent self-reported Impulse Control and Conscientiousness factors, indicating that better Common EF is associated with slightly better impulse control and higher conscientiousness. Furthermore, Malanchini et al. reported minimal genetic and environmental overlap between the task-based Common EF factor and the questionnaire-based Impulse Control and Conscientiousness factors. Finally, Malanchini et al. found that Common EF, Impulse Control, and Conscientiousness independently predicted academic outcomes. In two adult twin samples, Friedman et al. found modest negative phenotypic and genetic relationships between a Common EF factor and five impulsivity facets (2020). Friedman et al. also observed that the Common EF factor and several impulsivity facets, particularly negative urgency, independently statistically predicted an Externalizing psychopathology factor. Meanwhile, multiple impulsivity facets, but not the Common EF factor, independently statistically predicted an Internalizing psychopathology factor. Taken together, these studies demonstrate that EFs and impulsivity are related but separate, and statistically predict psychopathology in adulthood and academic outcomes in childhood.

## Present Study

As reviewed in the prior sections, existing research suggests that EFs and impulsivity may be separable from each other across development and may both confer transdiagnostic risk for psychopathology in adulthood. However, it is unclear if EFs and impulsivity are independent transdiagnostic correlates of psychopathology in childhood. Breakdowns in control in childhood likely manifest differently than in adulthood (i.e., being disruptive in class, temper tantrums, biting). Additionally, the bottom-up processing of rewards component develops more quickly than the top-down control component of the dual systems model (Steinberg, 2010; Shulman et al., 2016), so relationships between these control-related constructs and psychopathology could vary across development. Therefore, we evaluate whether the patterns of phenotypic, genetic, and environmental relationships between EFs, impulsivity, and psychopathology found in adult samples hold in sample of children. Specifically, we address the following three questions: (1) To what extent are these different control-related constructs, EFs and impulsivity, relate to each other during middle childhood? (2) To what extent are EFs and impulsivity related to psychopathology during middle childhood? (3) And do EFs and impulsivity share genetic and environmental influences with each other and psychopathology in middle childhood?

We use a large national sample of over 11,000 children from the Adolescent Brain Cognitive Development*^SM^* (ABCD) Study, including a subset of 749 twin pairs, to examine these three main questions using a previously published task-based latent variable model of EFs (Freis et al., 2022), self-reported Urgency, Planning (lack of), Perseverance (lack of), Sensation Seeking, Positive Urgency, Impulsive Behavior Scale (UPPS-P) (Barch et al., 2018), as well parent-reported psychopathology as measured by the Child Behavior Checklist (CBCL) (Achenbach, 2009).

## Materials and Methods

### Sample

The ABCD study^1^ is comprised of 11,875 children (ages 9–10.9, *M* = 9.91, *SD* = 0.62, 48% female) recruited from sites located across 21 United States. The ABCD study was strategically recruited to be demographically (52.2% White; 15.1% Black; 20.4% Hispanic; 3.2% Asian, American Indian/Alaska Native, or Native Hawaiian and other Pacific Islander; 9.2% Multiple races selected) and socioeconomically (Annual family income < $25K 16.1%; $25K–$49K 15.1%; $50K–74K 14.0%; $75K–99K 14.1%; $100K–199K 29.5%; $200K+11.2%) diverse and generally match the American Community Survey national estimates for demographic characteristics (Garavan et al., 2018; Heeringa and Berglund, 2020). Four ABCD study sites are dedicated to twin-pair recruitment (Iacono et al., 2018). Phenotypic analyses used data from the entire available baseline sample, while the genetically informed analyses used data from 749 same-sex twin pairs (329 monozygotic [MZ], 420 dizygotic [DZ]). We classified ABCD twin pair zygosities using genetic data by computing the probability of identity by descent (IBD), the proportion of segregating alleles shared across the genome. We classified twin pairs with IBD estimates between 0.4 and 0.7 as DZ (fraternal) and pairs with estimates > 0.9 as MZ (identical).

### Measures

#### Executive Functions

The ABCD data-collection protocol includes National Institutes of Health (NIH) Toolbox tasks measuring several cognitive domains including EF, memory, language, and processing speed (Bleck et al., 2013; Gershon et al., 2013; Hodes et al., 2013). We used an existing published model of EF in the ABCD study (Freis et al., 2022) based on the well replicated unity/diversity model of EFs (Friedman and Miyake, 2017) which includes flanker, card sort, and list sort tasks from the NIH Toolbox^2^, and the emotional *n*-back and stop signal (SST) tasks. Task reliabilities have been established and described in pilot ABCD data and previous studies (Casey et al., 2018; Luciana et al., 2018).

In the ABCD study, all NIH Toolbox tasks are behavioral tasks and administered via iPads. The NIH Toolbox tasks described here were administered in succession in laboratory settings at each ABCD study site at baseline. *List Sort* measures working memory. Participants are presented with pictures of animals and foods of different sizes. Each picture is also accompanied by the animal or food name presented aloud by the iPad. Participants have to say the list back, but in size order. List sort begins with only a single category, then participants are presented with a two-item list. If the two-item trial is answered correctly the list length increases. The maximum list length is seven items, and the final score is the total number of correct responses (Tulsky et al., 2013, 2014; Luciana et al., 2018). *Flanker* measures interference control and attention. Participants focus on a middle arrow and indicate which direction the arrow is pointing while ignoring surrounding arrows pointing in the same or opposite direction. In congruent trials all arrows point in the same direction, while in incongruent trials the arrows point in the opposite direction of the center arrow. Participants indicate the direction of the center arrow by pressing a button on an iPad. This version of the flanker includes 4 blocks with 25 task trials per block. *Card Sort* measures cognitive flexibility. Participants are shown objects and asked to match them to two shapes at the bottom of the screen based on a particular rule (color or shape) This version of the card sort includes 40 total task trials (including 30 mixed-block trials). For both Flanker and Card Sort, the final standard scores provided by the Toolbox scoring algorithm account for accuracy and reaction time: Scores were created using a two-vector procedure that incorporated accuracy for all participants and reaction time for participants that maintained high accuracy (>80% accurate); trials with reaction times lower than 100 milliseconds or reaction times greater than 3 SDs from the participants’ mean reaction time were screened as outliers (Zelazo et al., 2013). More details on scoring procedures and measures derived from the NIH Toolbox tasks are detailed in Zelazo et al. (2013), Luciana et al. (2018).

*Emotional n-back* (Cohen et al., 2016) was administered during fMRI scanning and measures working memory and emotional reactivity. Participants match stimuli based on a previously seen target that is either 2 back or 0 back. Each of 2 runs includes 40 trials (Casey et al., 2018). The dependent measure was overall task accuracy in the 2 back trials.

The *SST* (Logan, 1994) was administered during fMRI scanning and measures response inhibition. Participants are presented with a leftward or rightward facing arrow and are instructed to indicate the direction of the arrow. Participants are asked to respond as quickly and as accurately as possible and are required to inhibit their response when presented with an unexpected upward-facing “stop” arrow. Each of 2 runs includes 180 trials (Casey et al., 2018). To measure stop-signal RT, we subtracted the mean stop-signal delay (the time between the start of the go trial and the stop trial), from the mean “go” trial RT. The emotional n-back and SST were administered in the scanner and participants responded via a button response box.

#### Impulsivity

The UPPS-P integrates multiple separable aspects of impulsivity into a single questionnaire (Lynam, 2013). Five facets of impulsivity were measured via child-report at the first wave of data collection using a shortened 20-item version of the UPPS-P scales (Barch et al., 2018) including negative urgency (α = 0.63), lack of planning (also known as lack of premeditation) (α = 0.73), sensation seeking (α = 0.49), positive urgency (α = 0.77), and lack of perseverance (α = 0.70). Children rated their agreement on a 1–4 scale on items including “When I feel bad, I often do things I later regret in order to make myself feel better now,” (negative urgency) and “I tend to stop and think before doing things,” (lack of planning, reverse coded). Children completed the UPPS-P under the supervision of a trained research assistant.

#### Psychopathology

Psychopathology symptoms were measured via parent-report at the first wave of data collection with the Achenbach Parent Report Child Behavior Checklist (CBCL) (Achenbach, 2009). Self-reported psychopathology was not available in the release of the dataset that we used. The CBCL is scored to assess eight scales intended to capture different dimensions of psychopathology symptoms: withdrawn/depressed, somatic problems, anxious/depressed, social problems, thought problems, attention problems, rule-breaking, and aggression. The CBCL scales have been reported to be reliable (withdrawn/depressed α = 0.80, somatic problems α = 0.78, anxious/depressed α = 0.84, social problems α = 0.82, thought problems α = 0.78, attention problems α = 0.86, rule-breaking α = 0.85, and aggression α = 0.94) (Achenbach, 2009). The withdrawn/depressed, somatic problems, and anxious/depressed scales can be summed to create an internalizing composite or used as indicators for an Internalizing factor, and the rule-breaking and aggression scales can be summed to create an externalizing composite or used as indicators for an Externalizing factor. In the present study, we used the scales as indicators for Internalizing and Externalizing factors.

### Statistical Procedures

We retrieved the data through the ABCD NIMH data archive portal. We used the pre-calculated composite scores and did not apply transformations or additional screening for flanker, list sort, and card sort. Data cleaning and screening procedures for the *n*-back and SST tasks are detailed in Freis et al. (2022). Ns and descriptives for each cognitive task and survey measures can be found in Table 1. We regressed out age and sex for all cognitive tasks and survey measures and used the standardized residuals in all models. All models were estimated in Mplus version 8 (Muthén and Muthén, 1998-2017). In the phenotypic models we used Mplus’ TYPE = COMPLEX to account for non-independence of children from the same families. Chi-square tests of model fit are sensitive to sample size, so we additionally assessed model fit as good with the following criteria: root mean square error of approximation (RMSEA) < 0.06 and confirmatory fit index (CFI) > 0.95 (Hu and Bentler, 1998). Finally, for the genetic models, the statistical significance of parameter estimates was assessed using chi-square difference tests, where a significant *p*-Value (*p* < 0.05) indicates a significant reduction in model fit, and therefore suggests a significant parameter was dropped. For reference, we also included bootstrapped confidence intervals from Mplus for the genetic and environmental correlations. These bootstrapped confidence intervals are an estimate, so if the confidence intervals and chi-square difference test disagreed, we used the difference test as our determination of significance.

**TABLE 1:** Descriptive statistics for cognitive tasks and survey measures.

Measure	*n*	*M*	*SD*	Min	Max	Range	Skew	Kurtosis
Flanker	11712	94	9.14	51	116	65	−1	1.49
List	11669	96.64	12.09	36	136	100	–0.54	0.87
Card	11713	92.52	9.51	50	120	70	–0.82	2.04
Nback	7938	0.75	0.12	0.15	1	0.85	–0.43	0.19
SST	8262	299.79	65.99	55.97	614.32	558.36	0.34	1
Negative Urgency	11849	8.49	2.65	4	16	12	0.28	–0.44
Lack of Planning	11849	7.74	2.38	4	16	12	0.72	0.78
Sensation Seeking	11849	9.77	2.68	4	16	12	0.04	–0.49
Positive Urgency	11849	7.99	2.96	4	16	12	0.47	–0.41
Lack of Perseverance	11849	7.04	2.25	4	16	12	0.87	1.01
ANX	11864	2.52	3.06	0	26	26	1.93	4.87
DEP	11864	1.03	1.71	0	15	15	2.48	7.82
SOM	11864	1.49	1.95	0	16	16	1.91	4.82
SOC	11864	1.62	2.28	0	18	18	2.12	5.61
THOUGHT	11864	1.62	2.2	0	18	18	2.26	6.88
ATT	11864	2.98	3.49	0	20	20	1.44	1.83
RB	11864	1.19	1.86	0	20	20	2.57	9.59
AGG	11864	3.26	4.35	0	36	36	2.18	6.07
INT Sum	11864	5.05	5.53	0	51	51	1.94	5.12
EXT Sum	11864	4.45	5.86	0	49	49	2.3	7.02

*Descriptive statistics for cognitive tasks after data screening (see Supplementary Table 1 for descriptive statistics before screening). List, list sort; Card, card sort; Nback, accuracy on 2-back trials; SST, stop signal RT, calculated by the mean “go” trial RT – the mean stop signal delay; ANX, anxious/depressed; DEP, withdrawn/depressed; SOM, somatic complaints; SOC, social problems; THOUGHT, thought problems; ATT, attention problems; RB, rule-breaking behavior; AGG, aggressive behavior; INT Sum, Internalizing composite made up by summing anxious/depressed, withdrawn/depressed, and somatic complaints. EXT Sum, Externalizing composite made up by summing rule-breaking behavior and aggressive behavior.*

The classical twin design (Rijsdijk and Sham, 2002) employed here uses covariances from identical (MZ) and fraternal (DZ) twins to decompose variation in an outcome into additive genetic (A), shared environmental (C; environmental influences that lead siblings to correlate, e.g., socioeconomic status), and non-shared environmental (E; environmental influences that lead siblings to not correlate, e.g., differential parental treatment or different friend groups) influences. E also includes measurement error for non-latent variables. MZ twins share 100% of their segregating genes (genes that vary between people), while DZ twins, like non-twin siblings, share on average 50% of their segregating genes. Both MZ and DZ twins share their familial environments.

Genetic influences (A) are implied when MZ twin pairs show a higher correlation for a trait compared to DZ twin pairs. The proportion of total variation in an outcome that is accounted for by genetic differences in a population is the heritability. Shared environmental (C) influences are implied when the DZ correlation is greater than half the MZ correlation, i.e., when DZ twins are more similar than would be expected just considering their degree of genetic similarity. Non-shared environmental influences (E) are those that lead twins to differ; thus, they are implied when the MZ correlation is less than 1.

The design can be extended to estimate genetic (*r*A) and environmental (*r*C and *r*E) correlations across outcomes, which are informed by cross-trait covariances within and across twins (Rijsdijk and Sham, 2002). For example, common genetic influences (genetic correlations) are implied when the cross-twin cross-trait covariances between scores on impulsivity questionnaires and psychopathology are higher in MZ pairs relative to DZ pairs. Both genetic and environmental correlations across traits contribute to their phenotypic correlation. The part of the phenotypic correlation explained by genetic overlap is the bivariate heritability, and the part explained by environmental overlap is bivariate environmentality (either shared or non-shared). We calculated bivariate heritability by multiplying the *r*A of two traits by the square roots of those traits’ heritability estimates. Similarly, we calculated bivariate environmentality by multiplying the *r*C or *r*E by the square roots of the C and E estimates, respectively.

## Results

### Preliminary Analyses: A Latent Model of Executive Function Factors and Behavioral Genetic Decompositions of Executive Function Factors and Impulsivity Facets

**FIGURE 1:**
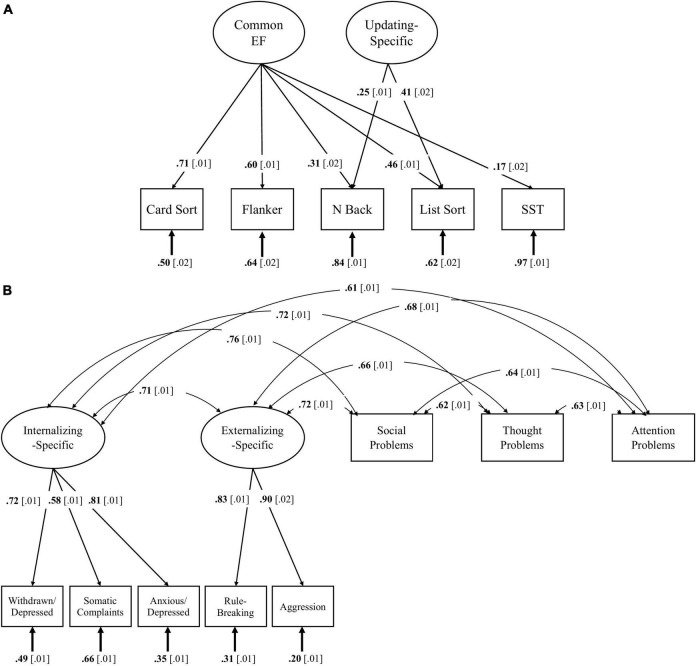
Phenotypic factor model of the unity/diversity model executive function (EF) originally presented in Freis et al. (2022) **(A)** and the correlated factors model of psychopathology **(B)**. Ellipses signify latent variables; rectangles signify observed variables. Numbers on single-headed arrows signify standardized factor loadings. Numbers in brackets are standard errors. Numbers at the ends of arrows are residual variances. Double headed arrows signify correlations. Solid lines and boldface type signify *p* < 0.05.

Before we could address the main study questions, we first had to derive a latent model of EF in the ABCD sample. Table 2 contains correlations between the EF tasks, impulsivity facets, and each CBCL composite. Our latent variable model of EFs included a Common EF factor representing performance across all EF tasks and an Updating-Specific factor representing performance specific to updating tasks (list sort and emotional *n*-back) after accounting for the influence of the Common EF factor. A phenotypic factor model of Common EF and an orthogonal Updating-Specific factor with loadings for *n*-back and list sort fit the data well χ^2^(4) = 43.48, *p* < 0.001 RMSEA = 0.029, CFI = 0.990; Δχ^2^(1) = 80.04, *p* < 0.001. This model is depicted in Figure 1A and details about fitting and identifying this EF model are in Freis et al. (2022).

**TABLE 2:** Correlation matrix for cognitive tasks and survey measures.

	1	2	3	4	5	6	7	8	9	10	11	12	13	14	15	16	17	18
(1) Flanker	1																	
(2) Card	**0.43**	1																
(3) List	**0.28**	**0.32**	1															
(4) Nback	**0.16**	**0.18**	**0.21**	1														
(5) SSTrt	−**0.07**	−**0.13**	−**0.07**	−**0.10**	1													
(6) Negative Urgency	−**0.04**	−**0.05**	−**0.06**	−**0.02**	0.00	1												
(7) Planning	**0.03**	–0.01	**0.03**	0.02	**0.03**	**0.15**	1											
(8) Sensation Seeking	**0.05**	**0.02**	**0.04**	**0.04**	**0.04**	**0.13**	**0.05**	1										
(9) Positive Urgency	−**0.07**	−**0.11**	−**0.13**	−**0.05**	**0.03**	**0.49**	**0.20**	**0.19**	1									
(10) Perseverance	−**0.03**	−**0.07**	−**0.06**	−**0.04**	**0.03**	**0.13**	**0.45**	−**0.10**	**0**.**17**	1								
(11) ATT	−**0.12**	−**0.14**	−**0.14**	−**0.09**	**0.04**	**0.13**	**0.16**	**0.02**	**0**.**14**	**0**.**21**	1							
(12) ANX	−**0.02**	−**0.03**	–0.01	−**0.02**	–0.01	**0.08**	**0.05**	−**0.04**	**0**.**03**	**0**.**09**	**0**.**48**	1						
(13) DEP	−**0.05**	−**0.06**	−**0.06**	−**0.07**	–0.01	**0.06**	**0.04**	−**0.05**	**0**.**04**	**0**.**10**	**0**.**45**	**0**.**58**	1					
(14) SOM	–0.01	−**0.02**	−**0.02**	−**0.03**	0.00	**0.03**	**0.03**	0.00	**0**.**03**	**0**.**07**	**0**.**36**	**0**.**48**	**0**.**40**	1				
(15) SOC	−**0.11**	−**0.13**	−**0.14**	−**0.09**	**0.03**	**0.11**	**0.09**	−**0.02**	**0**.**10**	**0**.**12**	**0**.**64**	**0**.**61**	**0**.**56**	**0**.**43**	1			
(16) THOUGHT	−**0.04**	−**0.06**	−**0.05**	−**0.04**	0.01	**0.09**	**0.10**	0.00	**0**.**07**	**0**.**12**	**0**.**63**	**0**.**58**	**0**.**50**	**0**.**44**	**0**.**61**	1		
(17) RB	−**0.07**	−**0.11**	−**0.13**	−**0.08**	0.00	**0.14**	**0.12**	**0.03**	**0**.**14**	**0**.**10**	**0**.**55**	**0**.**37**	**0**.**40**	**0**.**30**	**0**.**56**	**0**.**51**	1	
(18) AGG	−**0.07**	−**0.09**	−**0.09**	−**0.08**	0.00	**0.16**	**0.14**	**0.02**	**0**.**12**	**0**.**11**	**0**.**62**	**0**.**54**	**0**.**50**	**0**.**39**	**0**.**66**	**0**.**60**	**0**.**74**	1

*Correlation matrix of cognitive tasks after data screening and survey measures. List, list sort; Card, card sort; Nback, accuracy on 2-back trials; SST, stop signal RT, calculated by the mean “go” trial RT – the mean stop signal delay; Planning, Lack of Planning; Perseverance, Lack of Perseverance; ANX, anxious/depressed; DEP, withdrawn/depressed; SOM, somatic complaints; SOC, social problems; THOUGHT, thought problems; ATT, attention problems; RB, rule-breaking behavior; AGG, aggressive behavior. Bold font = p < 0.05.*

Armed with a reasonable latent model of EF, we then went on to examine the degree to which these EF factors appear to be influenced by genetic as compared to environmental factors. In behavioral genetic model of EF, 71% of the variance in Common EF was attributable to A (Δχ^2^(1) = 10.87, *p* = 0.001), 4% was attributable to C Δχ^2^(1) = 0.05, *p* = 0.823, and 25% was attributable to E (Δχ^2^(1) = 11.70, *p* < 0.01). In the same model, 48% of the variance in Updating-Specific was attributable to A (Δχ^2^(1) = 0.24, *p* = 0.626), 52% was attributable to C Δχ^2^(1) = 0.24, *p* = 0.625, and 0% was attributable to E (Δχ^2^(1) = 0.00, *p* = 1.00).

For the impulsivity facets, we observed small to moderate estimates of A (10–40%), minimal estimates of C (0–14%), and large estimates of E (60–78%, which includes measurement error for non-latent variables). See Table 3 for twin correlations, estimated variance components, and model fit for univariate ACE models for each impulsivity facet.

**TABLE 3:** Twin correlations, variance components, and model fit indices for univariate ACE models of cognitive tasks and survey measures.

	Twin correlations		Variance components			Model fit				
Measure	MZ	DZ	A	C	E	χ^2^	df	*p*	RMSEA	CFI
Flanker	**0.26**	0.09	**0.23**	0.00	**0.77**	11.09	6	0.086	0.048	0.786
List Sort	**0.43**	**0.25**	**0.27**	0.13	**0.60**	19.55	6	0.003	0.078	0.851
Card Sort	**0.38**	**0.13**	**0.35**	0.00	**0.65**	2.48	6	0.877	0.000	1.00
Stop Signal	**0.36**	**0.21**	**0.31**	0.05	**0.64**	6.57	6	0.363	0.017	0.984
N-Back	**0.31**	0.13	**0.30**	0.00	**0.70**	7.77	6	0.256	0.030	0.901
Negative Urgency	**0.28**	**0.17**	0.19	0.08	**0.74**	5.59	6	0.471	0.000	1.00
Lack of Planning	**0.26**	0.03	**0.22**	0.00	**0.78**	4.02	6	0.674	0.000	1.00
Sensation Seeking	**0.27**	**0.16**	0.27	0.02	**0.72**	6.98	6	0.323	0.021	0.971
Positive Urgency	**0.25**	**0.18**	0.10	0.14	**0.76**	6.16	6	0.406	0.008	0.995
Lack of Perseverance	**0.44**	**0.12**	**0.40**	0.00	**0.60**	4.90	6	0.557	0.000	1.00
Anxious/Depressed	**0.46**	**0.29**	0.19	**0.23**	**0.58**	19.63	6	0.003	0.078	0.879
Withdrawn/depressed	**0.53**	0.09	**0.48**	0.00	**0.52**	15.62	6	0.016	0.065	0.912
Somatic Problems	**0.55**	**0.35**	0.14	**0.35**	**0.52**	52.91	6	0.000	0.144	0.724
Social Problems	**0.54**	**0.31**	**0.58**	0.00	**0.42**	23.23	6	0.001	0.088	0.885
Thought Problems	**0.53**	**0.29**	**0.56**	0.00	**0.44**	45.73	6	0.000	0.133	0.721
Attention Problems	**0.67**	**0.18**	**0.66**	0.00	**0.34**	31.57	6	0.000	0.107	0.873
Rule-Breaking	**0.59**	**0.34**	**0.64**	0.00	**0.36**	39.62	6	0.000	0.122	0.824
Aggression	**0.70**	**0.39**	**0.73**	0.00	**0.28**	48.54	6	0.000	0.138	0.854
Internalizing Sum	**0.59**	**0.37**	**0.24**	**0.30**	**0.46**	23.04	6	0.001	0.087	0.914
Externalizing Sum	**0.71**	**0.42**	**0.74**	0.00	**0.26**	58.22	6	0.000	0.152	0.831

*Bold font, significant parameter. Bold twin correlations indicate p < 0.05. Significance of variance components tested with χ^2^ model comparison tests. A, additive genetic variance; C, shared environmental variance; E, non-shared environmental variance.*

### Question 1: To What Extent Do Executive Functions and Impulsivity Relate to Each Other During Middle Childhood?

To examine the associations between our control-related constructs we first examined relationships between latent variables of EFs and the composite scores of the five impulsivity facets. At the phenotypic level, EFs and impulsivity were weakly and inconsistently related (*r*s = –0.15 to 0.06, all *p*s < 0.05 except for Common EF with lack of planning *p* = 0.774) (Table 4).

**TABLE 4:** Correlations between latent common executive function (EF), updating-specific, and impulsivity facets.

	1	2	3	4	5	6	7
(1) Common EF	1						
(2) Updating-Specific	−	1					
(3) Positive Urgency	−**0.14** [0.01]	−**0.15** [0.02]	1				
(4) Negative Urgency	−**0.07** [0.01]	−**0.07** [0.02]	**0.49** [0.01]	1			
(5) Planning	0.00 [0.01]	**0.06** [0.02]	**0.20** [0.01]	**0.15** [0.01]	1		
(6) Perseverance	−**0.08** [0.01]	−**0.05** [0.02]	**0.17** [0.01]	**0.13** [0.01]	**0.45** [0.01]	1	
(7) Sensation Seeking	**0.04** [0.01]	**0.06** [0.02]	**0.19** [0.01]	**0.13** [0.01]	**0.05** [0.01]	−**0.11** [0.01]	1

*Planning, Lack of Planning. Perseverance, Lack of Perseverance. Numbers in brackets are standard errors. Bold font indicates p < 0.05. Dashes indicate a correlation that was not estimated. N = 11,870. See Supplementary Materials for results of phenotypic models in the twin subsample.*

Though we did not observe many significant phenotypic correlations between EFs and the impulsivity facets, we nonetheless calculated genetic and environmental correlations between Common EF, Updating-Specific, and each impulsivity facet to examine if EFs and impulsivity were more closely related at the genetic or environmental levels. We observed minimal significant genetic and environmental correlations between Common EF, Updating-Specific, and the five impulsivity facets (Table 5). Genetic correlations between Common EF and the impulsivity facets ranged from –0.15 to 0.29, and the only significant genetic correlation was between Common EF and sensation seeking (*r*A = 0.29, Δχ^2^(1) = 7.69, *p* = 0.006). Genetic correlations between Updating-Specific and the impulsivity facets ranged from –0.66 to 0.35, but none of these correlations were significant according to chi-square comparison tests. We did not estimate correlations between variance components estimated at or near 0 due to model convergence issues, and many of the C variance components we observed were at or near 0. Therefore, we only estimated one shared environmental correlation between Updating-Specific and positive urgency, however this correlation was not significant (*r*C = 0.29, Δχ^2^(1) = 0.17, *p* = 0.678). Non-shared environmental correlations between Common EF and the impulsivity facets ranged from –0.20 to 0.01., and the only significant non-shared environmental correlation was between Common EF and positive urgency (*r*E = –0.20, Δχ^2^(1) = 4.84, *p* = 0.028). We did not estimate non-shared environmental correlations between Updating-Specific and the impulsivity composites because the E estimate for Updating-Specific was 0.

**TABLE 5:** Genetic and Non-shared environmental correlations of executive function latent variables and UPPS-subscales.

	Common EF				Updating-specific			
	A = 71%, C = 4%, E = 25%				A = 48%, C = 52%, E = 0%			
	rA	*r*E	Biv A	Biv E	*r*A	*r*E	Biv A	Biv E
**Negative Urgency**								
A = 19%, C = 8%, **E = 73%**	–0.09 [–0.59,0.26]	0.01 [–0.21,0.22]	–0.03	0.00	0.00 [–0.63,0.60]	–	0.00	–
**Lack of Planning**								
**A = 22%**, C = 0%, **E = 78%**	0.16 [–0.18,0.50]	–0.03 [–0.20,0.16]	0.06	–0.01	0.35 [0.02, > 1]	–	0.13	–
**Sensation Seeking**								
A = 26%, C = 2%, **E = 72%**	**0.29** [0.09,0.76]	–0.12 [–0.34,0.07]	0.13	–0.05	0.06 [–0.43,0.54]	–	0.02	–
**Positive Urgency**								
A = 10%, C = 14%, **E = 76%**	–0.15 [–0.76,0.26]	–**0.20** [–0.43, –0.01]	–0.04	–0.09	–0.66 [ < –1, > 1]	–	–0.16	–
**Lack of Perseverance**								
**A = 40%**, C = 0%, **E = 60%**	–0.13 [–0.38,0.08]	–0.07 [–0.26,0.16]	–0.07	–0.03	–0.03 [–0.38,0.34]	–	–0.02	–

*Bold font = significant parameter tested with χ^2^ model comparison test. Biv A, bivariate heritability. Biv E, bivariate non-shared environmentality. Numbers in brackets are bootstrapped confidence intervals from Mplus. Dashes indicate a parameter that was not estimated. We only estimate one shared environmental correlation (between positive urgency and Updating-Specific), and for space did not include the rC and bivariate shared environmentality in the Table (rC = 0.29 [<–1, >1]). We did not estimate non-shared environmental correlations for Updating-Specific because the E estimate 0.*

### Question 2: To What Extent Are Executive Functions and Impulsivity Related to Psychopathology During Middle Childhood?

The correlated factors model of psychopathology that we used to address question 2 is shown in Figure 1B. It includes an Externalizing factor with rule-breaking and aggressive behavior as indicators; an Internalizing factor with withdrawn/depressed, somatic problems, and anxious/depressed as indicators; and the attention problems, social problems, and thought problems composites as separate constructs (correlated with but not indicators of the Externalizing and Internalizing factors). This model fit the data well χ^2^(14) = 441.66, *p* < 0.001 RMSEA = 0.051, CFI = 0.983. We based this model on the CBCL scoring guide, so we only had two Externalizing indicators. Therefore, we constrained the loadings to be equal to improve stability of the Externalizing factor (Kline, 2016).

**FIGURE 2:**
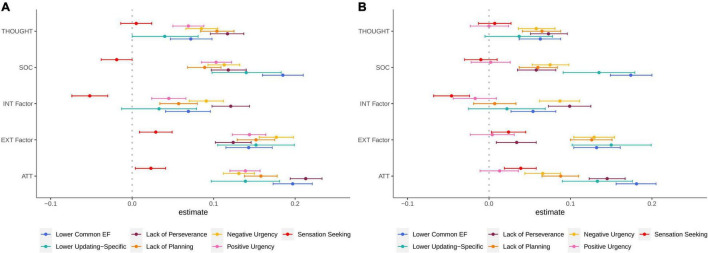
Phenotypic correlations with panel **(A)** and regression coefficients for panel **(B)** relationships between EFs, impulsivity, and a correlated factors model of psychopathology. The correlation and regression coefficients estimates are on the *X*-axis. Psychopathology is on the *Y*-axis. SOC, social problems; THOUGHT, thought problems; ATT, attention problems; INT Factor, factor with anxious/depressed, withdrawn/depressed, and somatic complaints as indicators; EXT Factor, Externalizing factor with rule-breaking behavior and aggressive behavior as indicators. Color of the lines and dots represent EF factors and impulsivity facets. Error bars are 95% Cls from Mplus. The metric of EF and Updating-Specific was reversed to be in the same metric as impulsivity where higher numbers indicate lower control. *N* = 11,873. See Supplementary Materials for results of phenotypic models in the twin subsample.

To address question 2, we added the EF factors and the five impulsivity facets to this correlated factors psychopathology model (Figure 2A). This model fit the data well χ^2^(14) = 441.66, *p* < 0.001 RMSEA = 0.051, CFI = 0.983. Higher psychopathology across symptom dimensions was consistently related to both lower Common EF and higher impulsivity across all facets except for sensation seeking (lower Common EF with psychopathology *r*s = –0.07 to –0.20; higher impulsivity facets with psychopathology *r*s = 0.05 to 0.18). Higher social problems, attention problems, and the Externalizing factor were related to lower Updating-Specific (*r*s = –0.14 to –0.15).

In structural equation model regressions that controlled for the correlations between the EF factors and impulsivity facets, higher psychopathology across domains remained significantly related to lower Common EF (Figure 2B). Higher social problems, attention problems, and the Externalizing factor remained significantly related to Lower Updating-Specific. Higher psychopathology across domains also remained significantly related to higher negative urgency and lack of perseverance. Externalizing and other psychopathology composites remained significantly related to lack of planning. Finally, psychopathology was no longer related to positive urgency, and psychopathology remained inconsistently and weakly related to sensation seeking (see Supplementary Table 2 for all correlations and regression coefficients of the full phenotypic model). Overall, Common EF and several impulsivity facets independently statistically predicting multiple aspects of psychopathology suggests task and ratings-based methods measure separable aspects of control that are related to broad psychopathology symptoms.

### Question 3: Do Executive Functions and Impulsivity Share Genetic and Environmental Influences With Each Other and Psychopathology in Middle Childhood?

The answer to question 3 informs whether if EFs and impulsivity relate to psychopathology for different reasons (shared genetic or non-shared environmental influences). To answer this question, we calculated genetic and environmental correlations between EFs, impulsivity, and psychopathology that were significant in the phenotypic model. Information on the genetic correlated factors model of psychopathology without genetic and environmental correlations between EFs and impulsivity can be found in Table 6 and the Supplementary Materials.

**TABLE 6:** Genetic and Non-shared environmental correlations of executive function latent variables, UPPS-subscales, and Correlated Factors model of psychopathology.

	EXT Factor		INT Factor		ATT		SOC		THOUGHT	
	A = 76%, C = 0%, E = 24%		A = 64%, C = 0%, E = 36%		A = 59%, C = 0%, E = 41%		A = 55%, C = 0%, E = 45%		A = 51%, C = 0%, E = 49%	
	*r*A	*r*E	*r*A	*r*E	*r*A	*r*E	*r*A	*r*E	*r*A	*r*E
Common EF	–**0.18**	0.06	–0.07	–0.04	–**0.25**	–0.18	–**0.24**	–0.09	–0.08	–0.04
**A = 71%**, C = 4%, **E = 25%**	[–0.42, –0.04]	[–0.22,0.34]	[–0.28,0.10]	[–0.31,0.16]	[–0.55, –0.09]	[–0.41,0.01]	[–0.54, –0.08]	[–0.30,0.09]	[–0.26,0.08]	[–0.22,0.14]
Updating–sp.	–0.16	–	–	–	–0.13	–	0.03	–	–	–
A = 48%, C = 52%, E = 0%	[–0.49,0.10]				[–0.39,0.18]		[–0.20,0.27]			
Neg. Urgency	**0.21**	**0.17**	0.03	**0.15**	**0.25**	0.06	0.13	0.08	0.00	**0.11**
A = 19%, C = 8%, **E = 73%**	[0.01,0.74]	[0.04,0.30]	[–0.20, 0.30]	[0.03,0.26]	[0.06,0.83]	[–0.04,0.15]	[–0.08,0.55]	[–0.02,0.17]	[–0.30,0.27]	[0.02,0.20]
Planning	**0.24**	**0.14**	**0.22**	–**0.14**	**0.27**	0.02	**0.20**	–0.01	**0.26**	0.02
**A = 22%**, C = 0%, **E = 78%**	[0.06,0.47]	[–0.01,0.27]	[0.01,0.52]	[–0.28, –0.02]	[0.05,0.57]	[–0.11,0.13]	[–0.02,0.45]	[–0.12,0.09]	[0.03,0.53]	[–0.08,0.12]
Sen. Seeking	–0.01	0.11	0.12	–**0.18**	–0.06	0.08	–	–	–	–
A = 26%, C = 2%, **E = 72%**	[–0.23,0.15]	[–0.02,0.22]	[–0.02,0.43]	[–0.29, –0.08]	[–0.30,0.08]	[–0.01,0.17]				
Pos. Urgency	**0.30**	0.01	0.02	–0.02	**0.42**	0.02	0.22	–0.01	0.09	0.02
A = 10%, C = 14%, **E = 76%**	[0.03, > 1]	[–0.10,0.13]	[–0.33,0.39]	[–0.15,0.12]	[0.10,>1]	[–0.08,0.12]	[–0.07,0.79]	[–0.09,0.09]	[–0.24,0.45]	[–0.05,0.11]
Perseverance	**0.18**	–0.01	**0.25**	–**0.16**	**0.27**	0.03	**0.19**	–0.04	**0.26**	–0.10
**A = 40%**, C = 0%, **E = 60%**	[0.03,0.33]	[–0.15,0.13]	[0.08,0.47]	[–0.29, –0.04]	[0.10,0.46]	[–0.09,0.14]	[–0.02,0.40]	[–0.18,0.08]	[0.08,0.45]	[–0.22,0.02]

*EXT Factor, Externalizing factor with rule-breaking behavior and aggressive behavior as indicators; INT Factor, Internalizing factor with anxious/depressed, withdrawn/depressed, and somatic complaints as indicators; ATT, attention problems; SOC, social problems; THOUGHT, thought problems; Updating-sp., Updating-Specific; Neg. Urgency, Negative Urgency; Planning, Lack of Planning; Sen. Seeking, Sensation Seeking; Pos. Urgency, Positive Urgency; Perseverance, Lack of Perseverance. Bold font = p < 0.05, according to a χ^2^ difference test. Numbers in brackets are bootstrapped confidence intervals from Mplus. Dashes indicate that the parameter was not estimated. We did not decompose non-significant phenotypic correlations (Figure 2A and Supplementary Table 2), and we did not estimate non-shared environmental correlations for Updating-Specific because the E estimate for this factor was 0.*

Cross-twin cross-trait correlations (Supplementary Tables 8, 9) did not suggest the presence of C and a model with *r*Cs between the EF and psychopathology factors would not converge, so we removed C variance components for the latent psychopathology factors and *r*Cs between the EF and psychopathology factors from the model. Additionally, we did not observe any estimates of C greater than 0 in the genetic correlated factors model of psychopathology model (except for somatic symptoms), so we dropped all psychopathology composite C estimates of 0 from the models and did not estimate shared environmental correlations to aid model convergence. Model fit for each genetic model and the results of all model comparisons can be found in Supplementary Tables 13–19. Genetic and non-shared environmental correlations between EFs, impulsivity, and psychopathology can be found in Table 6 along with bootstrapped confidence intervals.

The Externalizing factor (*r*A = –0.18, Δχ^2^(1) = 6.45, *p* = 0.01), attention problems (*r*A = –0.25, Δχ^2^(1) = 10.18, *p* = 0.001), and social problems (*r*A = –0.24, Δχ^2^(1) = 9.60, *p* = 0.002) were negatively genetically related to Common EF. The psychopathology factors and composites were not significantly genetically related to Updating-Specific. Moreover, we did not observe significant non-shared environmental correlations between any of the psychopathology factors or composites and Common EF.

We observed varied genetic and non-shared environmental correlations between the correlated factors model of psychopathology and impulsivity. The Externalizing factor (*r*A = 0.21, Δχ^2^(1) = 4.93, *p* = 0.03) and attention problems composite (*r*A = 0.25, Δχ^2^(1) = 5.87, *p* = 0.02) displayed significant positive genetic correlations with negative urgency. Additionally, the Internalizing factor (*r*E = 0.15, Δχ^2^(1) = 6.07, *p* = 0.02), Externalizing factor (*r*E = 0.17, Δχ^2^(1) = 7.91, *p* = 0.005), and thought problems scale (*r*E = 0.11, Δχ^2^(1) = 5.30, *p* = 0.02) displayed significant positive non-shared environmental correlations with negative urgency. We observed significant positive genetic correlations between all psychopathology factors and composites (*r*As = 0.20–0.27) and lack of planning. Internalizing (*r*E = –0.14, Δχ^2^(1) = 5.84, *p* = 0.02) and Externalizing (*r*E = 0.14, Δχ^2^(1) = 4.80, *p* = 0.03) were related to lack of planning in different directions at the non-shared environmental level. The psychopathology factors or composites were not genetically related to sensation seeking. However, the Internalizing factor displayed a significant non-shared environmental correlation with sensation seeking (*r*E = –0.18, Δχ^2^(1) = 11.46, *p* = 0.001). Externalizing (*r*A = 0.30, Δχ^2^(1) = 5.36, *p* = 0.02) and attention problems (*r*A = 0.42, Δχ^2^(1) = 8.86, *p* = 0.003) were significantly positively related to positive urgency at the genetic level, and unrelated to all psychopathology factors and composites at the non-shared environmental level. Finally, all psychopathology factors and composites were positively genetically correlated with lack of perseverance (*r*As = 0.18–0.27). Internalizing displayed a negative non-shared environmental correlation with lack of perseverance (*r*E = –0.16, Δχ^2^(1) = 7.17, *p* = 0.001). Overall, though we observed genetic correlations between Common EF and psychopathology, and between some impulsivity facets and psychopathology, these results indicate that impulsivity, but not EFs, displayed environmental associations with psychopathology.

We also examined phenotypic, genetic, and environmental relationships between EFs, impulsivity, and the individual CBCL composite scores as well as between EFs, impulsivity, and a bifactor *p*-factor model and observed similar results. The *p*-factor, indicating transdiagnostic risk for psychopathology, was negatively related to Common EF at the phenotypic and genetic levels. The *p*-factor was also positively related to lack of planning and lack of perseverance at the phenotypic and genetic levels. The Internalizing-Specific factor was positively related to Common EF at the phenotypic level, which is inconsistent with the results of the correlated factors model of psychopathology. However, unlike the Internalizing-Specific factor, the Internalizing factor in the correlated factors model includes *p*-factor variance. These results along with the cross-twin cross trait correlations between the latent EF variables and bifactor psychopathology model can be found in the Supplementary Materials.

## Discussion

We examined phenotypic, genetic, and environmental relationships between task-based latent variables of EFs, child self-reported impulsivity ratings, and multiple dimensions of parent-reported psychopathology in a large, demographically diverse childhood sample to better understand relationships between control-related constructs and psychopathology in this age group. First, we examined phenotypic relationships between EFs and impulsivity and found that these sometimes-conflated constructs assessed via different methods were not strongly related to each other. Second, we calculated genetic and environmental correlations between EFs and impulsivity to see if these constructs were more related at the genetic or environmental levels and again found few significant relationships. Third, we examined phenotypic relationships between EFs, impulsivity, and multiple domains of psychopathology and found that after accounting for the correlations of the EF factors with the impulsivity facets, Common EF, negative urgency, and lack of perseverance were independently related to all psychopathology factors and composites. Finally, informed by our phenotypic results, we examined genetic and environmental correlations between EF, impulsivity, and psychopathology and identified significant genetic overlap between Common EF and several symptom dimensions of psychopathology. Meanwhile, we observed significant genetic and non-shared environmental overlap between several impulsivity facets and different dimensions of psychopathology.

EFs and impulsivity are sometimes described as two ends of the same spectrum or equated as measures of the broad construct of self-regulation (Bickel et al., 2012; Nigg, 2017). However, our results fit in with a growing body of literature that indicates that EFs measured with tasks and impulsivity measured with ratings are not closely related at the phenotypic, genetic, or environmental levels (Harden et al., 2017; Malanchini et al., 2018; Friedman et al., 2020). Because latent variable modeling minimizes measurement error, observing weak and inconsistent associations between latent variables of EFs and impulsivity measures indicates that the explanation for these low correlations may not be exclusively due to low reliability in EF tasks. These low correlations remain even when researchers have examined relationships between different measurement methods of a theoretically single construct. For example, Snyder et al. (2020) observed weak associations between a task-based latent variable of Common EF and a self-report based latent variable of Common EF using the Behavior Rating Inventory of Executive Function–Self-Report (BRIEF-SR), further reinforcing the possibility that task-based and rating-based measures may tap different aspects of control.

Methodological considerations beyond reliability could be important in understanding why these different measures may tap different constructs. For example, tasks assess control over short intervals, while ratings ask about typical behavior. Tasks are usually administered in highly controlled laboratory settings and assess performance under optimal conditions, which may not translate to real world contexts that are full of distractions (Samyn et al., 2015; Friedman and Gustavson, 2022). Moreover, ratings may ask about emotional contexts (e.g., urgency items in the UPPS-P questionnaire), whereas EF tasks typically do not incorporate emotionally salient stimuli. One potentially promising avenue for research on control-related constructs and their correlates is designing more emotionally valenced EF tasks that are applicable to real world contexts. For example, one study that employed more emotionally valenced tasks have found slightly higher correlations with self-reported impulsivity (Verdejo-Garcia et al., 2021). Overall, more research focusing on what factors distinguish tasks and ratings is needed to better understand the constructs that they measure.

Both lower EFs and higher impulsivity have been proposed as risk factors for numerous aspects of psychopathology (Nigg, 2017). In our study, Common EF and several impulsivity facets were consistently related to multiple symptom dimensions of psychopathology in the correlated factors model, and to the *p*-factor in the bifactor model. These findings across modeling strategies indicate that lower Common EF, higher negative urgency, and higher lack of perseverance appear to be transdiagnostic correlates of increased psychopathology. That is, breakdowns in Common EF and these aspects of impulse control may confer risk for a broad range of psychopathology dimensions. For example, breakdowns in Common EF, higher negative urgency, and lack of perseverance may manifest as difficulty regulating aggressive and rule-breaking behavior, attention, thoughts, and behavior in social situations.

In the *p*-factor model, the relationship between Common EF and the Internalizing-Specific factor was positive and opposite in direction from the association observed between Common EF and the Internalizing factor in the correlated factors model of psychopathology. Prior studies employing multiple models of psychopathology have reported such flipped directionality of correlations with specific factors in bifactor *p*-factor models (Caspi et al., 2014; Clark et al., 2021). Such patterns suggest that although the variance in internalizing that is shared with other aspects of psychopathology is negatively related to Common EF, the variance unique to internalizing is positively related to Common EF. This pattern could mean that higher internalizing problems that are separate from transdiagnostic risk for psychopathology are related to better Common EF. However, this relationship should be interpreted with caution as specific factors in bifactor models can be unstable, idiosyncratic, and difficult to interpret (van Bork et al., 2017; Watts et al., 2019).

Importantly, Common EF, negative urgency, and lack of perseverance all remained phenotypically significantly statistically predictive of all aspects of the correlated factors model of psychopathology and the *p*-factor in the bifactor model in a multiple regression. These results indicate that Common EF and some facets of impulsivity account for different variance in multiple aspects of psychopathology. This finding is important because it confirms that the separability of EFs and impulsivity (or more generally the separability of task-based and rating-based measures of control) is not simply a consequence of low reliability or validity of one of the measure types. Rather, it appears that their unique variances are “interesting,” in that they tap different variance in psychopathology (Friedman and Banich, 2019). Thus, Common EF and impulsivity could be important to jointly consider when examining research questions related to control abilities and psychopathology. Moreover, elucidating the key dimensions that distinguish these control-related measures may yield insight into the nature of self-regulation problems associated with psychopathology.

Our results are consistent with existing work in adult samples that found that EFs and impulsivity are distinct but are both broadly associated with multiple forms of psychopathology (Friedman et al., 2020), suggesting that a similar pattern is evident earlier in the development of psychopathology. Additionally, our findings are consistent with existing literature on EFs, impulsivity, and academic outcomes in childhood (Malanchini et al., 2018) in that EFs and impulsivity are distinct, and both associated with behavioral and psychological outcomes.

Breakdowns in cognitive and behavioral control manifesting as lower Common EF and higher impulsivity in childhood may be crucial to understanding developmental psychopathology. It could be that breakdowns in control lead to behavioral problems and psychopathology symptoms directly and causally. For example, disruption of neural systems that support the development of EFs in childhood may lead to an increased risk for general psychopathology (Zelazo, 2020). On the other hand, breakdowns in control and psychopathology symptoms could share similar genetic and environmental etiologies. Overall, though our cross-sectional study does not elucidate the directionality of the relationship between lower Common EF, higher impulsivity, and more psychopathology symptoms, our findings do emphasize that both Common EF and impulsivity are important to consider in studies of control and psychopathology. Additionally, this work suggests that measures of the dual systems model are not interchangeable even in childhood.

### Interpreting Behavioral Genetic Models

We used behavioral genetic models to examine genetic and environmental correlations between EFs, impulsivity, and psychopathology. Behavioral genetic models are valuable due to their ability to examine relationships between traits at different levels of analysis and assess to what extent phenotypic relationships may differ from genetic and environmental relationships. For example, these models allow for the examination of relationships between traits while removing unique environmental influences, including measurement error, and enable us to evaluate the extent to which relationships between traits may reflect shared familial risk factors.

First, consistent with existing work with adult samples (Friedman et al., 2020), we found low genetic overlap between EFs and impulsivity in this middle childhood sample. This result suggests low overlap in individual differences assessed by these two self-regulation related constructs, even when removing variance due to measurement error and environmental influences that may be specific to EFs or impulsivity. Second, also similar to relationships observed in adults (Friedman et al., 2020), we identified significant genetic correlations between Common EF and several dimensions of psychopathology as well as significant genetic and non-shared environmental correlations between several impulsivity scales and different dimensions of psychopathology. These findings indicate that low Common EF and high impulsivity may confer genetic risk for psychopathology transdiagnostically in multiple stages of development. Considered in light with the low genetic and environmental overlap between Common EF and impulsivity dimensions, these results suggest that the genetic risk for psychopathology conferred by low Common EF and high impulsivity is different.

The A and E estimates for Common EF (high A and small but significant E), psychopathology scales and factors (moderate to high A estimates and moderate E estimates) and impulsivity scales (low A estimates and high E) were broadly consistent with prior work in children, adolescents, and adults (Engelhardt et al., 2015; Harden et al., 2017, 2020; Friedman et al., 2020). The high E estimates for the impulsivity scales may be partially attributable to measurement error, however, considering the high reliability for most UPPS-P scales, it is unlikely that the high E estimate is exclusively due to measurement error.

Existing genetically informed work has lacked racial and ethnic diversity, potentially limiting the generalizability of findings from twin data. Though the twin subsample is less demographically representative than the entire ABCD sample (for example, 66% White vs. 52% White), these genetically informed findings may be more generalizable to children in the United States than results from studies with less demographically diverse samples.

Importantly, these significant genetic influences and correlations should not be interpreted in a genetically deterministic manner. High heritability estimates do not imply that these traits are genetically determined or immutable. Heritability is an estimate of the proportion of variation is an outcome, like Common EF or an attention problems composite, that is accounted for by genetic differences in a specific population, at a particular time, and under particular environmental conditions. Heritability estimates can change across time and environmental contexts (Tucker-Drob et al., 2013; Friedman et al., 2016, 2021).

### Limitations and Future Directions

There are several methodological limitations to this study. First, the ABCD cognitive battery was not designed to tap the unity/diversity model of EFs and did not have enough EF tasks to construct more than the Common EF and Updating-Specific EF factors. Additionally, our Updating-Specific factor only had two indicators, as did the Externalizing factor in the psychopathology model. Factors with only two indicators can be prone to technical issues like difficulty with model estimation and instability (Kline, 2016). Finally, the scoring algorithms for the NIH Toolbox tasks provide overall indices of performance, which could make these measures slightly more related to general cognitive ability than traditional EF tasks, potentially altering relationships with impulsivity and psychopathology.

The impulsivity facets were measured via self-report with a short form of the UPPS. The reliability estimates of the impulsivity facets measured by Cronbach’s alpha were generally acceptable, but not high. The 9-and-10-year-olds responding to the UPPS questionnaire in this study may not have the best insight into their impulsivity. Finally, psychopathology symptoms were parent-reported. Although the CBCL is commonly used to measure child psychopathology, parents may not have full insight into their children’s psychopathology symptoms (Huang, 2017).

Though the ABCD Study is ongoing and longitudinal, this study was cross-sectional and used data from the baseline ABCD protocol. One important open question in the literature relates to the directionality of the relationships between EFs, impulsivity, and psychopathology. For example, breakdowns in EFs or other forms of control could lead to the development of multiple dimensions of psychopathology. Additionally, lower EF and control could be consequences of higher psychopathology. One study used longitudinal ABCD data and different modeling strategies than our project to examine prospective relationships between an EF factor and a *p* factor. These researchers found that EF was both a risk factor for and a consequence of higher transdiagnostic psychopathology (Romer and Pizzagalli, 2021). However, in adolescence, others have found that higher internalizing and externalizing predict later EF deficits, but not the other way around, indicating lower EF may be a consequence of psychopathology (Donati et al., 2021). Overall, these mixed findings in different age groups suggest that the directionality of the relationships between breakdowns in control and higher psychopathology remains unclear and is an avenue for future research. This question could be particularly interesting to examine across development and before and after the onset of puberty where psychopathology symptoms generally increase (Mendle, 2014). The longitudinal nature of ABCD will be valuable for better understanding how phenotypic, genetic, and environmental relationships between control-related constructs like EFs and impulsivity with psychopathology develop and change over time.

## Conclusion

Our findings in a large and diverse childhood sample extend two patterns present in self-regulation research: (1) task-based (EFs) and self-report measures (impulsivity scales) of control display weak and inconsistent relationships, suggesting separability and (2) EFs and some facets of impulsivity are independently associated with psychopathology across symptom dimensions (i.e., transdiagnostically). These findings indicate that the separability of these control related constructs and their relevance to behavioral and psychological outcomes may be consistent for different age groups and outcomes. Additionally, these results emphasize the importance of incorporating multiple measures of control in self-regulation studies. Using multiple measures may be critical to better understand relationships between control deficits and psychopathology.

## Data Availability Statement

Publicly available datasets were analyzed in this study. This data can be found here: The ABCD data repository grows and changes over time. The ABCD data used in this report came from ABCD release 2.0 (DOI: 10.15154/1503209, Study-specific NDA DOI: 10.15154/1524243). ABCD data is publicly available.

## Ethics Statement

The studies involving human participants were reviewed and approved by all ABCD procedures were approved by a central institutional review board and comply with the World Medical Association Declaration of Helsinki and APA ethical standards. The University of California San Diego institutional review board has stated that analyses using publicly released ABCD data are not human subjects research and do not require their own protocol approval. Written informed consent to participate in this study was provided by the participants’ legal guardian/next of kin.

## Author Contributions

SF conducted the data analysis and drafted the manuscript, under the supervision of NF. All authors provided critical revisions to the manuscript and contributed to the conception of the work.

## Author Disclaimer

This manuscript reflects the views of the authors and may not reflect the opinions or views of the NIH or ABCD consortium investigators.

## Conflict of Interest

The authors declare that the research was conducted in the absence of any commercial or financial relationships that could be construed as a potential conflict of interest.

## Publisher’s Note

All claims expressed in this article are solely those of the authors and do not necessarily represent those of their affiliated organizations, or those of the publisher, the editors and the reviewers. Any product that may be evaluated in this article, or claim that may be made by its manufacturer, is not guaranteed or endorsed by the publisher.

## Footnotes

1
https://abcdstudy.org

2
https://www.healthmeasures.net/explore-measurement-systems/nih-toolbox
