# Supplementary material for: Executive Functions and Impulsivity as Transdiagnostic Correlates of Psychopathology in Childhood: A Behavioral Genetic Analysis
Source: Front Hum Neurosci. 2022 Mar 25;16:863235. doi: 10.3389/fnhum.2022.863235 (PMC9012075; doi:10.3389/fnhum.2022.863235)
Supplement: Supplementary file 1 [file Data_Sheet_1.docx]

Supplementary Material

**Executive functions and impulsivity as transdiagnostic correlates of psychopathology in childhood: A behavioral genetic analysis**

**Samantha M. Freis^1,2^, Claire L. Morrison^1,2^, Harry R. Smolker^3^, Marie T. Banich^2, 3^, Roselinde H. Kaiser^2, 3,4^, John K. Hewitt^1,2^, Naomi P. Friedman^1,2^**

^1^Institute for Behavioral Genetics, University of Colorado Boulder.

^2^Department of Psychology and Neuroscience, University of Colorado Boulder.

^3^Institute of Cognitive Science, University of Colorado Boulder.

^4^ Renee Crown Wellness Institute, University of Colorado Boulder.

*** Correspondence:**Samantha M. Freis
Samantha.freis@colorado.edu

**Supplemental Table 1**

*Descriptive Statistics for Cognitive Tasks & Survey Measures Before Data Screening*

| Measure | *n* | *M* | *SD* | Min | Max | Range | Skew | Kurtosis |
| --- | --- | --- | --- | --- | --- | --- | --- | --- |
| Flanker | 11712 | 94 | 9.14 | 51 | 116 | 65 | -1 | 1.49 |
| List | 11669 | 96.64 | 12.09 | 36 | 136 | 100 | -0.54 | 0.87 |
| Card | 11713 | 92.52 | 9.51 | 50 | 120 | 70 | -0.82 | 2.04 |
| NBack2 | 9468 | 0.71 | 0.17 | 0 | 1 | 1 | -1.04 | 1.49 |
| SST | 9598 | 299.69 | 85.07 | -692 | 702.69 | 1394.69 | -1.23 | 9.5 |

*Note*. Descriptive statistics for cognitive tasks before data screening. List = list sort; Card = card sort; NBack = accuracy on 2-back trials; SST = stop signal RT, calculated by the mean “go” trial RT - the mean stop signal delay.

**Supplemental Table 2**

*Regressions & Correlations from Full Phenotypic Model of EFs, Impulsivity, and Correlated Factors Psychopathology Model*

|  | Regression coefficients for | | | | |  |  |
| --- | --- | --- | --- | --- | --- | --- | --- |
| Independent Variable | EXT | INT | Attention Probs | Social Probs | Thought Probs | |  |
| Common EF | **-.13** [.01] | **-.05** [.01] | **-.18** [.01] | **-.17** [.01] | **-.06** [.01] | |  |
| Updating-Specific | **-.15** [.03] | -.02 [.02] | **-.13** [.02] | **-.14** [.02] | -.04 [.02] | |  |
| Negative Urgency | **.13** [.01] | **.09** [.01] | **.07** [.01] | **.08** [.01] | **.06** [.01] | |  |
| Lack of Planning | **.13** [.01] | .01 [.01] | **.09** [.01] | **.06** [.01] | **.07** [.01] | |  |
| Sensation Seeking | .02 [.01] | **-.05** [.01] | **.04** [.01] | -.01 [.01] | .01 [.01] | |  |
| Positive Urgency | .00 [.01] | -.02 [.01] | .01 [.01] | .00 [.01] | .00 [.01] | |  |
| Lack of Perseverance | **.03** [.01] | **.10** [.01] | **.15** [.01] | **.06** [.01] | **.07** [.01] | |  |
|  | Correlations with | | | | | | |
| Independent Variable | EXT | INT | Attention Probs | Social Probs | Thought Probs | |  |
| Common EF | **-.14** [.01] | **-.07** [.01] | **-.20** [.01] | **-.19** [.01] | **-.07** [.01] | |  |
| Updating-Specific | **-.15** [.02] | -.03 [.02] | **-.14** [.02] | **-.14** [.02] | -.04 [.02] | |  |
| Negative Urgency | **.18** [.01] | **.09** [.01] | **.13** [.01] | **.11** [.01] | **.09** [.01] | |  |
| Lack of Planning | **.15** [.01] | **.06** [.01] | **.16** [.01] | **.09** [.01] | **.10** [.01] | |  |
| Sensation Seeking | **.03** [.01] | **-.05** [.01] | **.02** [.01] | -.02 [.01] | .01 [.01] | |  |
| Positive Urgency | **.14** [.01] | **.05** [.01] | **.14** [.01] | **.10** [.01] | **.07** [.01] | |  |
| Lack of Perseverance | **.12** [.01] | **.12** [.01] | **.21** [.01] | **.12** [.01] | **.12** [.01] | |  |

*Note*. INT = Internalizing factor with anxious/depressed, withdrawn/depressed, and somatic complaints as indicators; EXT = Externalizing factor with rule-breaking behavior and aggressive behavior as indicators. Numbers in brackets are standard errors. Bold font indicates *p* < .05.

**Supplemental Table 3**

*Regressions & Correlations from Full Phenotypic Model of EFs, Impulsivity, and Correlated Factors Psychopathology Model in the Twin Subsample*

|  | Regression coefficients for | | | | |
| --- | --- | --- | --- | --- | --- |
| Independent Variable | EXT | INT | Attention Probs | Social Probs | Thought Probs |
| Common EF | -.09 [.05] | -.02 [.05] | **-.17** [.04] | **-.14** [.04] | -.04 [.04] |
| Updating-Specific | -.19 [.11] | -.07 [.11] | -.15 [.09] | -.12 [.08] | -.11 [.09] |
| Negative Urgency | **.14** [.04] | **.11** [.04] | **.08** [.03] | **.09** [.03] | **.07** [.03] |
| Lack of Planning | **.18** [.05] | .01 [.05] | **.10** [.04] | **.08** [.04] | **.12** [.04] |
| Sensation Seeking | .00 [.03] | **-.09** [.03] | -.01 [.03] | **-.06** [.03] | -.03 [.03] |
| Positive Urgency | -.03 [.04] | -.05 [.04] | .01 [.04] | -.02 [.03] | -.03 [.04] |
| Lack of Perseverance | -.01 [.04] | .02 [.04] | **.08** [.03] | .00 [.03] | .00 [.03] |

|  | Correlations with | | | | |  |
| --- | --- | --- | --- | --- | --- | --- |
| Independent Variable | EXT | INT | Attention Probs | Social Probs | Thought Probs | |
| Common EF | -.08 [.05] | -.03 [.05] | **-.17** [.04] | **-.14** [.04] | -.04 [.04] | |
| Updating-Specific | -.15 [.10] | -.07 [.11] | -.14 [.08] | -.11 [.08] | -.09 [.08] | |
| Negative Urgency | **.15** [.03] | **.08** [.03] | **.11** [.03] | **.09** [.02] | **.07** [.03] | |
| Lack of Planning | **.15** [.04] | .00 [.04] | **.10** [.03] | **.06** [.03] | **.10** [.03] | |
| Sensation Seeking | -.01 [.03] | **-.10** [.03] | -.02 [.03] | **-.07** [.03] | -.04 [.03] | |
| Positive Urgency | **.09** [.03] | .00 [.03] | **.11** [.03] | .05 [.03] | .03 [.03] | |
| Lack of Perseverance | **.09** [.04] | .05 [.03] | **.14** [.03] | **.07** [.03] | **.06** [.03] | |

*Note*. INT = Internalizing factor with anxious/depressed, withdrawn/depressed, and somatic complaints as indicators; EXT = Externalizing factor with rule-breaking behavior and aggressive behavior as indicators. Numbers in brackets are standard errors. Bold font indicates *p* < .05.

**EFs, Impulsivity, and Psychopathology Composites**

We examined phenotypic correlations between Common EF, Updating-Specific, the impulsivity facets, and the eight psychopathology composite scores from the CBCL: withdrawn/depressed, somatic problems, anxious/depressed, social problems, thought problems, attention problems, rule breaking, and aggression. This model fit the data well, χ^2^(42) = 153.01, *p* < 0.001 RMSEA = 0.015, CFI = 0.998. All results from this model can be found in Supplemental Table 4.

**Supplemental Table 4**

*Regressions Coefficients from Full Phenotypic Model of EFs, Impulsivity, and CBCL Composites*

|  | Regression coefficients for | | | | | | | |
| --- | --- | --- | --- | --- | --- | --- | --- | --- |
| Independent Variable | ATT | ANX | DEP | SOM | SOC | THT | RB | AGG |
| Common EF | **-.18** [.01] | **-.03** [.01] | **-.07** [.01] | -.02 [.01] | **-.17** [.01] | **-.06** [.01] | **-.13** [.01] | **-.11** [.01] |
| Updating | **-.13** [.02] | .02 [.02] | **-.05** [.03] | -.02 [.02] | **-.13** [.03] | -.03 [.02] | **-.16** [.03] | **-.10** [.03] |
| Neg. Urgency | **.07** [.01] | **.09** [.01] | **.06** [.01] | .02 [.01] | **.08** [.01] | **.06** [.01] | **.08** [.01] | **.13** [.01] |
| Planning | **.09** [.01] | .01 [.01] | .01 [.01] | .00 [.01] | **.06** [.01] | **.06** [.01] | **.10** [.01] | **.11** [.01] |
| Sen. Seeking | **.04** [.01] | **-.04** [.01] | **-.04** [.01] | .01 [.01] | -.01 [.01] | .01 [.01] | **.03** [.01] | .01 [.01] |
| Pos. Urgency | .02 [.01] | -.02 [.01] | -.02 [.01] | .01 [.01] | .01 [.01] | .00 [.01] | .03 [.01] | .00 [.01] |
| Perseverance | **.15** [.01] | **.07** [.01] | **.08** [.01] | **.06** [.01] | **.06** [.01] | **.07** [.01] | **.03** [.01] | **.03** [.01] |

*Note*. Updating- Updating-Specific; Neg. Urgency = Negative Urgency; Planning = Lack of Planning; Sen. Seeking = Sensation Seeking. Pos. Urgency = Positive Urgency. Perseverance = Lack of Perseverance; ATT = attention problems; ANX = anxious/depressed; DEP = withdrawn/depressed; SOM = somatic complaints; SOC = social problems; THT = thought problems; RB = rule-breaking behavior; AGG = aggressive behavior. Bold font = *p* < .05. Numbers in brackets are standard errors.

**Supplemental Table 5**

*Correlations from Full Phenotypic Model of EFs, Impulsivity, and CBCL Composites*

|  | Correlations with | | | | | | | |
| --- | --- | --- | --- | --- | --- | --- | --- | --- |
| Independent Variable | ATT | ANX | DEP | SOM | SOC | THT | RB | AGG |
| Common EF | **-.20** [.01] | **-.04** [.01] | **-.09** [.01] | **-.03** [.01] | **-.19** [.01] | **-.07** [.01] | **-.14** [.01] | **-.12** [.01] |
| Updating | **-.13** [.02] | .01 [.02] | **-.06** [.03] | -.03 [.02] | **-.13** [.03] | -.04 [.02] | **-.16** [.03] | **-.10** [.03] |
| Neg. Urgency | **.13** [.01] | **.08** [.01] | **.06** [.01] | **.03** [.01] | **.11** [.01] | **.09** [.01] | **.14** [.01] | **.16** [.01] |
| Planning | **.16** [.01] | **.05** [.01] | **.05** [.01] | **.03** [.01] | **.09** [.01] | **.10** [.01] | **.12** [.01] | **.14** [.01] |
| Sen. Seeking | **.02** [.01] | **-.04** [.01] | **-.05** [.01] | .00 [.01] | -.02 [.01] | .01 [.01] | **.03** [.01] | **.02** [.01] |
| Pos. Urgency | **.14** [.01] | **.03** [.01] | **.04** [.01] | **.04** [.01] | **.10** [.01] | **.07** [.01] | **.14** [.01] | **.12** [.01] |
| Perseverance | **.21** [.01] | **.09** [.01] | **.11** [.01] | **.07** [.01] | **.12** [.01] | **.12** [.01] | **.10** [.01] | **.11** [.01] |

*Note*. Updating- Updating-Specific; Neg. Urgency = Negative Urgency; Planning = Lack of Planning; Sen. Seeking = Sensation Seeking. Pos. Urgency = Positive Urgency. Perseverance = Lack of Perseverance; ATT = attention problems; ANX = anxious/depressed; DEP = withdrawn/depressed; SOM = somatic complaints; SOC = social problems; THT = thought problems; RB = rule-breaking behavior; AGG = aggressive behavior. Bold font = *p* < .05. Numbers in brackets are standard errors.

**EFs, Impulsivity, and the *P*-factor**

We also estimated a bifactor *p*-factor model and allowed the orthogonal Externalizing-Specific and Internalizing-Specific factors to correlate. This model also fit the data well: χ^2^(15) = 574.02, *p* < 0.001 RMSEA = 0.056, CFI = 0.978.

**Supplemental Table 6**

*Regressions & Correlations from Full Phenotypic Model of EFs, Impulsivity, and P-factor Model*

|  | Regression coefficients for | | | Correlations with | | |
| --- | --- | --- | --- | --- | --- | --- |
| Independent  Variable | EXT | INT | *P* Factor | EXT | INT | *P* Factor |
| Common EF | **.05** [.02] | **.24** [.02] | **-.18** [.01] | **.05** [.02] | **.22** [.02] | **-.19** [.01] |
| Updating | **-.07** [.03] | **.23** [.04] | **-.13** [.02] | -.06 [.03] | **.21** [.03] | **-.14** [.02] |
| Neg. Urgency | **.11** [.02] | .03 [.02] | **.09** [.01] | **.12** [.01] | **-.05** [.02] | **.14** [.01] |
| Planning | **.10** [.02] | **-.15** [.02] | **.09** [.01] | **.06** [.02] | **-.14** [.02] | **.14** [.01] |
| Sen. Seeking | **.03** [.02] | **-.11** [.02] | .01 [.01] | **.06** [.01] | **-.10** [.02] | .00 [.01] |
| Pos. Urgency | .00 [.02] | **-.04** [.02] | .00 [.01] | **.06** [.01] | **-.14** [.02] | **.13** [.01] |
| Perseverance | **-.13** [.02] | -.02 [.02] | **.11** [.01] | **-.07** [.02] | **-.09** [.02] | **.18** [.01] |

*Note*. INT = Internalizing factor with anxious/depressed, withdrawn/depressed, and somatic complaints as indicators; EXT = Externalizing factor with rule-breaking behavior and aggressive behavior as indicators. Updating- Updating-Specific; Neg. Urgency = Negative Urgency; Planning = Lack of Planning; Sen. Seeking = Sensation Seeking. Pos. Urgency = Positive Urgency. Perseverance = Lack of Perseverance. Bold font = *p* < .05. Numbers in brackets are standard errors.

**Supplemental Table 7**

*Regressions & Correlations from Full Phenotypic Model of EFs, Impulsivity, and P-factor Model in the Twin Subsample*

|  | Regression coefficients for | | | Correlations with | | |
| --- | --- | --- | --- | --- | --- | --- |
| Independent  Variable | EXT | INT | *P* Factor | EXT | INT | *P* Factor |
| Common EF | .06 [.06] | **.20** [.07] | **-.14** [.05] | .08 [.06] | **.19** [.07] | **-.15** [.05] |
| Updating | -.11 [.13] | .11 [.13] | -.15 [.10] | -.07 [.12] | .07 [.12] | -.13 [.09] |
| Neg. Urgency | **.11** [.05] | .09 [.06] | **.10** [.03] | **.10** [.04] | .01 [.05] | **.11** [.03] |
| Planning | **.15** [.06] | **-.17** [.07] | **.12** [.05] | **.12** [.05] | **-.16** [.05] | **.10** [.04] |
| Sen. Seeking | .07 [.04] | -.08 [.05] | -.05 [.03] | **.09** [.04] | -.08 [.04] | -.06 [.03] |
| Pos. Urgency | -.05 [.05] | -.11 [.06] | -.01 [.04] | .04 [.04] | **-.15** [.04] | **.08** [.03] |
| Perseverance | -.06 [.06] | -.02 [.05] | .03 [.04] | -.01 [.05] | **-.10** [.04] | **.11** [.03] |

*Note*. INT = Internalizing factor with anxious/depressed, withdrawn/depressed, and somatic complaints as indicators; EXT = Externalizing factor with rule-breaking behavior and aggressive behavior as indicators. Updating- Updating-Specific; Neg. Urgency = Negative Urgency; Planning = Lack of Planning; Sen. Seeking = Sensation Seeking. Pos. Urgency = Positive Urgency. Perseverance = Lack of Perseverance. Bold font = *p* < .05. Numbers in brackets are standard errors.

**Supplemental Table 8**

*Cross-twin Cross-trait, Within-twin Cross-trait, and Cross-twin Within-trait Correlations between EFs and the Correlated Factors Model of Psychopathology for Monozygotic Twins*

| **MZ** | T1  EF | T1 UPD | T1 INT | T1 EXT | T1 ATT | T1 SOC | T1 THT | T2  EF | T2 UPD | T2 INT | T2 EXT | T2 ATT | T2 SOC | T2 THT |
| --- | --- | --- | --- | --- | --- | --- | --- | --- | --- | --- | --- | --- | --- | --- |
| T1 EF | - | - |  |  |  |  |  |  |  |  |  |  |  |  |
| T1 UPD | - | - |  |  |  |  |  |  |  |  |  |  |  |  |
| T1 INT | -.03 | -.08 | - |  |  |  |  |  |  |  |  |  |  |  |
| T1 EXT | -.08 | -.13 | **.71** | - |  |  |  |  |  |  |  |  |  |  |
| T1 ATT | **-.18** | -.11 | **.56** | **.66** | - |  |  |  |  |  |  |  |  |  |
| T1 SOC | **-.14** | -.09 | **.75** | **.67** | **.63** | - |  |  |  |  |  |  |  |  |
| T1 THT | -.03 | -.09 | **.69** | **.63** | **.57** | **.59** | - |  |  |  |  |  |  |  |
| T2 EF | .74 | - | -.02 | -.10 | **-.15** | **-.14** | .02 | - |  |  |  |  |  |  |
| T2 UPD | - | **1.00** | -.09 | -.16 | -.19 | -.06 | **-.28** | - | - |  |  |  |  |  |
| T2 INT | -.02 | -.09 | **.65** | **.66** | **.51** | **.50** | **.54** | -.03 | -.08 | - |  |  |  |  |
| T2 EXT | -.10 | -.16 | **.66** | **.79** | **.55** | **.56** | **.49** | -.08 | -.13 | **.71** | - |  |  |  |
| T2 ATT | **-.15** | -.19 | **.51** | **.55** | **.69** | **.55** | **.57** | **-.18** | -.11 | **.56** | **.66** | - |  |  |
| T2 SOC | **-.14** | -.06 | **.50** | **.56** | **.55** | **.59** | **.45** | **-.14** | -.09 | **.75** | **.67** | **.63** | - |  |
| T2 THT | .02 | **-.28** | **.54** | **.49** | **.46** | **.45** | **.53** | -.03 | -.09 | **.69** | **.63** | **.57** | **.59** | - |

*Note.* T1 = Twin 1; T2 = Twin 2; EF = Common EF; UPD = Updating-Specific; INT = Internalizing factor with anxious/depressed, withdrawn/depressed, and somatic complaints as indicators; EXT = Externalizing factor with rule-breaking behavior and aggressive behavior as indicators; ATT = attention problems; SOC = social problems; THT = thought problems. Dashes indicated correlations that were not estimated. Bold font = *p* < .05.

**Supplemental Table 9**

*Cross-twin Cross-trait, Within-twin Cross-trait, and Cross-twin Within-trait Correlations between EFs and the Correlated Factors Model of Psychopathology for Dizygotic Twins*

| **DZ** | T1 EF | T1 UPD | T1 INT | T1 EXT | T1 ATT | T1 SOC | T1 THT | T2 EF | T2 UPD | T2 INT | T2 EXT | T2 ATT | T2 SOC | T2 THT |
| --- | --- | --- | --- | --- | --- | --- | --- | --- | --- | --- | --- | --- | --- | --- |
| T1 EF | - | - |  |  |  |  |  |  |  |  |  |  |  |  |
| T1 UPD | - | - |  |  |  |  |  |  |  |  |  |  |  |  |
| T1 INT | -.03 | -.08 | - |  |  |  |  |  |  |  |  |  |  |  |
| T1 EXT | -.08 | -.13 | **.71** | - |  |  |  |  |  |  |  |  |  |  |
| T1 ATT | **-.18** | -.11 | **.56** | **.66** | - |  |  |  |  |  |  |  |  |  |
| T1 SOC | **-.14** | -.09 | **.75** | **.67** | **.63** | - |  |  |  |  |  |  |  |  |
| T1 THT | -.03 | -.09 | **.69** | **.63** | **.57** | **.59** | - |  |  |  |  |  |  |  |
| T2 EF | **.44** | - | .04 | -.01 | .03 | .00 | -.02 | - |  |  |  |  |  |  |
| T2 UPD | - | .88 | -.09 | .05 | .13 | .05 | .15 | - | - |  |  |  |  |  |
| T2 INT | .04 | -.09 | **.49** | **.50** | **.41** | **.39** | **.35** | -.03 | -.08 | - |  |  |  |  |
| T2 EXT | -.01 | .05 | **.50** | **.45** | **.36** | **.41** | **.32** | -.08 | -.13 | **.71** | - |  |  |  |
| T2 ATT | .03 | .13 | **.41** | **.36** | **.19** | **.26** | **.23** | **-.18** | -.11 | **.56** | **.66** | - |  |  |
| T2 SOC | .00 | .05 | **.39** | **.41** | **.26** | **.31** | **.32** | **-.14** | -.09 | **.75** | **.67** | **.63** | - |  |
| T2 THT | -.02 | .15 | **.35** | **.32** | **.23** | **.32** | **.28** | -.03 | -.09 | **.69** | **.63** | **.57** | **.59** | - |

*Note.* T1 = Twin 1; T2 = Twin 2; EF = Common EF; UPD = Updating-Specific; INT = Internalizing factor with anxious/depressed, withdrawn/depressed, and somatic complaints as indicators; EXT = Externalizing factor with rule-breaking behavior and aggressive behavior as indicators; ATT = attention problems; SOC = social problems; THT = thought problems. Dashes indicated correlations that were not estimated.

**Supplemental Table 10**

*Cross-twin Cross-trait, Within-twin Cross-trait, and Cross-twin Within-trait Correlations between EFs and the P-Factor Model for Monozygotic Twins*

| **MZ** | T1 EF | T1 UPD | T1 INT | T1 EXT | T1 P | T2 EF | T2 UPD | T2 INT | T2 EXT | T2 P |
| --- | --- | --- | --- | --- | --- | --- | --- | --- | --- | --- |
| T1 EF | - |  |  |  |  |  |  |  |  |  |
| T1 UPD | - | - |  |  |  |  |  |  |  |  |
| T1 INT | **.22** | -.01 | - |  |  |  |  |  |  |  |
| T1 EXT | .08 | -.09 | **-.20** | - |  |  |  |  |  |  |
| T1 P | **-.15** | -.09 | - | - | - |  |  |  |  |  |
| T2 EF | **.73** | - | **.18** | .01 | **-.12** | - |  |  |  |  |
| T2 UPD | - | **1.00** | .08 | .02 | -.17 | - | - |  |  |  |
| T2 INT | **.18** | .08 | **.27** | **.23** | - | **.22** | -.01 | - |  |  |
| T2 EXT | .01 | .02 | **.23** | **.70** | - | .08 | -.09 | **-.20** | - |  |
| T2 P | **-.12** | -.17 | - | - | **.80** | **-.15** | -.09 | - | - | - |

*Note.* T1 = Twin 1; T2 = Twin 2; EF = Common EF; UPD = Updating-Specific; INT = Internalizing-specific factor with anxious/depressed, withdrawn/depressed, and somatic complaints as indicators; EXT = Externalizing-specific factor with rule-breaking behavior and aggressive behavior as indicators; P = Common psychopathology factor. Dashes indicated correlations that were not estimated.

**Supplemental Table 11**

*Cross-twin Cross-trait, Within-twin Cross-trait, and Cross-twin Within-trait Correlations between EFs and the P-Factor Model for Dizygotic Twins*

| **DZ** | T1 EF | T1 UPD | T1 INT | T1 EXT | T1 P | T2 EF | T2 UPD | T2 INT | T2 EXT | T2 P |
| --- | --- | --- | --- | --- | --- | --- | --- | --- | --- | --- |
| T1 EF | - |  |  |  |  |  |  |  |  |  |
| T1 UPD | - | - |  |  |  |  |  |  |  |  |
| T1 INT | .22 | -.01 | - |  |  |  |  |  |  |  |
| T1 EXT | .08 | -.09 | -.20 | - |  |  |  |  |  |  |
| T1 P | -.15 | -.09 | - | - | - |  |  |  |  |  |
| T2 EF | .73 | - | .11 | .01 | -.02 | - |  |  |  |  |
| T2 UPD | - | .89 | -.36 | -.09 | .13 | - | - |  |  |  |
| T2 INT | .11 | -.36 | .11 | .09 | - | **.22** | **-.36** | - |  |  |
| T2 EXT | .01 | -.09 | .09 | .01 | - | .08 | -.09 | **-.20** | - |  |
| T2 P | -.02 | .13 | - | - | **.53** | **-.15** | -.09 | - | - | - |

*Note.* T1 = Twin 1; T2 = Twin 2; EF = Common EF; UPD = Updating-Specific; INT = Internalizing-specific factor with anxious/depressed, withdrawn/depressed, and somatic complaints as indicators; EXT = Externalizing-specific factor with rule-breaking behavior and aggressive behavior as indicators; P = Common psychopathology factor. Dashes indicated correlations that were not estimated.

**Supplemental Table 12**

*Genetic and Nonshared Environmental Correlations of EF Latent variables, UPPS-subscales, and P-Factor Model of Psychopathology*

|  | *P* Factor | | EXT-Specific | | INT-Specific | |
| --- | --- | --- | --- | --- | --- | --- |
|  | A = 60%, C = 21%, E = 19% | | A = 58%, C = 0%, E = 42% | | A = 25%, C = 0%, E = 75% | |
|  | rA | *r*E | *r*A | *r*E | *r*A | *r*E |
| Common EF |  |  |  |  |  |  |
| **A = 71%**, C = 4%, **E = 25%** | **-.19** [-.54, -.04] | -.21 [-.52, .08] | .00 [-.24, .27] | .23 [-.12, .65] | **.40** [.08, > 1] | .14 [-.26, .43] |
| Updating-Specific |  |  |  |  |  |  |
| A = 48%, C = 52%, E = 0% | -.37 [-.96, .05] | - | - | - | .08 [-1, > 1] | - |
| Negative Urgency |  |  |  |  |  |  |
| A = 19%, C = 8%, **E = 73%** | .14 [-.12, .54] | **.17** [.02, .29] | .18 [-.20, .86] | .08 [-.09, .25] | -.25 [-.35, > 1] | .06 [-.10, .22] |
| Lack of Planning |  |  |  |  |  |  |
| **A = 22%**, C = 0%, **E = 78%** | **.26** [.02, .66] | .04 [-.12, .20] | .04 [-.38, .36] | **.17** [.01, .35] | -.05 [-.65, .67] | **-.22** [-.40, -.05] |
| Sensation Seeking |  |  |  |  |  |  |
| A = 26%, C = 2%, **E = 72%** | - | - | -.04 [-.55, .26] | .15 [-.03, .32] | .41 [< -1, .12] | **-.27** [-.45, -.10] |
| Positive Urgency |  |  |  |  |  |  |
| A = 10%, C = 14%, **E = 76%** | .05 [-.69, .83] | .04 [-.10, .19] | .18 [-.36, .89] | .00 [-.18, .16] | -.80 [< -1, .05] | -.05 [-.22, .13] |
| Lack of Perseverance |  |  |  |  |  |  |
| **A = 40%**, C = 0%, **E = 60%** | **.27** [.05, .58] | .07 [-.01, .27] | -.10 [-.44, .16] | -.06 [-.27, .14] | .02 [-.46, .55] | **-.16** [-.37, -.01] |

*Note.* Bold font = significant parameter tested with $\chi$^2^ model comparison test. Numbers in brackets are bootstrapped confidence intervals from Mplus. Dashes indicate a parameter that was not estimated. We did not estimate nonshared environmental correlations for Updating-Specific because the E estimate 0.

**Supplemental Table 13**

*Model Comparisons from Independent EF and Correlated Psychopathology Genetic Models*

|  | Model Fit | | | | | Model fit vs. full Model | | |
| --- | --- | --- | --- | --- | --- | --- | --- | --- |
| **Model** | χ^2^ | *df* | *p* | RMSEA | CFI | $\Delta$χ^2^ | *df* | *p* |
| ACE: EF & UPD | 185.75 | 100 | < .001 | .048 | .872 |  |  |  |
| Without cEF | 185.80 | 101 | < .001 | .047 | .874 | .05 | 1 | .823 |
| Without eEF | 197.45 | 101 | < .001 | .050 | .860 | 11.70 | 1 | .001 |
| Without aEF | 196.62 | 101 | < .001 | .050 | .858 | 10.87 | 1 | .001 |
| Without aEF & cEF | 250.77 | 102 | < .001 | .062 | .779 | 65.02 | 2 | < .001 |
| Without cUPD | 185.99 | 101 | < .001 | .047 | .874 | .24 | 1 | .625 |
| Without eUPD | 185.75 | 101 | < .001 | .047 | .874 | .00 | 1 | 1.000 |
| Without aUPD | 185.99 | 101 | < .001 | .047 | .874 | .24 | 1 | .626 |
| Without aUPD & cUPD | 214.63 | 102 | < .001 | .054 | .832 | 28.88 | 1 | < .001 |
| ACE: Correlated Factors Psychopathology | 912.66 | 244 | < .001 | .086 | .899 |  |  |  |
| Without rA INT w/ EXT | 1211.39 | 245 | < .001 | .103 | .854 | 298.73 | 1 | < .001 |
| Without rA INT w/ Att | 1129.09 | 245 | < .001 | .098 | .867 | 216.43 | 1 | < .001 |
| Without rA INT w/ Soc | 1086.63 | 245 | < .001 | .096 | .873 | 173.97 | 1 | < .001 |
| Without rA INT w/ Thought | no convergence | |  |  |  |  |  |  |
| Without rA EXT w/ Att | 1136.11 | 245 | < .001 | .099 | .866 | 223.45 | 1 | < .001 |
| Without rA EXT w/ Soc | 1161.08 | 245 | < .001 | .100 | .862 | 248.42 | 1 | < .001 |
| Without rA EXT w/ Thought | 1077.50 | 245 | < .001 | .095 | .874 | 164.84 | 1 | < .001 |
| Without rA Att w/ Soc | 1087.05 | 245 | < .001 | .096 | .873 | 174.39 | 1 | < .001 |
| Without rA Att w/ Thought | 1059.06 | 245 | < .001 | .094 | .877 | 146.40 | 1 | < .001 |
| Without rA Soc w/ Thought | 1086.66 | 245 | < .001 | .096 | .873 | 174.00 | 1 | < .001 |

*Note.* A=additive genetic influences, C=shared environmental influences, E=nonshared environmental influences. EF=Common EF; UPD=Updating-Specific; EXT = Externalizing factor with rule-breaking behavior and aggressive behavior as indicators; INT = Internalizing factor with anxious/depressed, withdrawn/depressed, and somatic complaints as indicators; Att = attention problems; Soc = social problems; Thought = thought problems. Indented models are nested within preceding non-indented models, $\Delta$χ^2^ *p* < .05 indicates a significant reduction in model fit and that a significant parameter was dropped from the model.

**Supplemental Table 14**

*Model Comparisons from Genetic Models of EFs Correlated with Impulsivity Facets*

|  | Model Fit | | | | | Model fit vs. full Model | | |
| --- | --- | --- | --- | --- | --- | --- | --- | --- |
| **Model** | χ^2^ | *df* | *p* | RMSEA | CFI | $\Delta$χ^2^ | *df* | *p* |
| ACE: EFs with Negative Urgency | 221.33 | 143 | < .001 | .038 | .888 |  |  |  |
| Without rA EF | 221.86 | 144 | < .001 | .038 | .889 | .53 | 1 | .465 |
| Without rA UPD | 221.33 | 144 | < .001 | .038 | .889 | .00 | 1 | 1.000 |
| Without rE EF | 221.34 | 144 | < .001 | .038 | .889 | .01 | 1 | .924 |
| ACE: EFs with Lack of Planning | 243.18 | 143 | < .001 | .043 | .860 |  |  |  |
| Without rA EF | 244.909 | 144 | < .001 | .043 | .859 | 1.73 | 1 | .188 |
| Without rA UPD | 246.772 | 144 | < .001 | .044 | .857 | 3.59 | 1 | .058 |
| Without rE EF | 243.265 | 144 | < .001 | .043 | .861 | .09 | 1 | .768 |
| ACE: EFs with Sensation Seeking | 262.72 | 143 | < .001 | .047 | .839 |  |  |  |
| Without rA EF | 270.405 | 144 | < .001 | .048 | .830 | 7.69 | 1 | .006 |
| Without rA UPD | 262.802 | 144 | < .001 | .047 | .841 | .09 | 1 | .771 |
| Without rE EF | 264.444 | 144 | < .001 | .047 | .838 | 1.73 | 1 | .189 |
| ACE: EFs with Positive Urgency | 223.43 | 142 | < .001 | .039 | .887 |  |  |  |
| Without rA EF | 224.329 | 143 | < .001 | .039 | .887 | .90 | 1 | .344 |
| Without rA UPD | 224.145 | 143 | < .001 | .039 | .887 | .71 | 1 | .399 |
| Without rC UPD | 223.61 | 143 | < .001 | .039 | .888 | .17 | 1 | .678 |
| Without rE EF | 228.268 | 143 | < .001 | .040 | .881 | 4.84 | 1 | .028 |
| ACE: EFs with Lack of Perseverance | 229.09 | 143 | < .001 | .040 | .886 |  |  |  |
| Without rA EF | 231.310 | 144 | < .001 | .04 | .884 | 2.22 | 1 | .136 |
| Without rA UPD | 229.131 | 144 | < .001 | .04 | .887 | .04 | 1 | .832 |
| Without rE EF | 229.672 | 144 | < .001 | .040 | .886 | .59 | 1 | .444 |

*Note.* A=additive genetic influences, C=shared environmental influences, E=nonshared environmental influences. EF=Common EF; UPD=Updating-Specific. Indented models are nested within preceding non-indented models, $\Delta$χ^2^ *p* < .05 indicates a significant reduction in model fit and that a significant parameter was dropped from the model.

**Supplemental Table 15**

*Model Comparisons from Genetic Models of EFs with Correlated Factors Psychopathology Model*

|  | Model Fit | | | | | Model fit vs. full Model | | |
| --- | --- | --- | --- | --- | --- | --- | --- | --- |
| **Model** | χ^2^ | *df* | *p* | RMSEA | CFI | $\Delta$χ^2^ | *df* | *p* |
| ACE: EFs with Correlated Factors Psychopathology | 1463.94 | 661 | < .001 | .057 | .892 |  |  |  |
| Without rA EF w/ INT | 1464.72 | 662 | < .001 | .057 | .892 | .78 | 1 | .377 |
| Without rA EF w/ EXT | 1470.38 | 662 | < .001 | .057 | .891 | 6.45 | 1 | .011 |
| Without rA EF w/ Att | 1474.12 | 662 | < .001 | .057 | .890 | 10.18 | 1 | .001 |
| Without rA EF w/ Soc | 1473.53 | 662 | < .001 | .057 | .890 | 9.60 | 1 | .002 |
| Without rA EF w/ Thought | 1464.90 | 662 | < .001 | .057 | .892 | .96 | 1 | .327 |
| Without rA UPD w/ EXT | 1464.77 | 662 | < .001 | .057 | .892 | .83 | 1 | .362 |
| Without rA UPD w/ Att | 1464.40 | 662 | < .001 | .057 | .892 | .46 | 1 | .497 |
| Without rA UPD w/ Soc | 1463.97 | 662 | < .001 | .057 | .892 | .04 | 1 | .852 |
| Without rE EF w/ INT | 1464.05 | 662 | < .001 | .057 | .892 | .12 | 1 | .735 |
| Without rE EF w/ EXT | 1464.18 | 662 | < .001 | .057 | .892 | .25 | 1 | .619 |
| Without rE EF w/ Att | 1466.99 | 662 | < .001 | .057 | .892 | 3.05 | 1 | .081 |
| Without rE EF w/ Soc | 1464.88 | 662 | < .001 | .057 | .892 | .94 | 1 | .332 |
| Without rE EF w/ Thought | 1464.09 | 662 | < .001 | .057 | .892 | .16 | 1 | .691 |

*Note.* A=additive genetic influences, C=shared environmental influences, E=nonshared environmental influences. EF=Common EF; UPD=Updating-Specific; EXT = Externalizing factor with rule-breaking behavior and aggressive behavior as indicators; INT = Internalizing factor with anxious/depressed, withdrawn/depressed, and somatic complaints as indicators; Att = attention problems; Soc = social problems; Thought = thought problems. Indented models are nested within preceding non-indented models, $\Delta$χ^2^ *p* < .05 indicates a significant reduction in model fit and that a significant parameter was dropped from the model.

**Supplemental Table 16**

*Model Comparisons from Genetic Models of Impulsivity Facets with Correlated Factors Psychopathology Model*

|  | Model Fit | | | | | Model fit vs. full Model | | |
| --- | --- | --- | --- | --- | --- | --- | --- | --- |
| **Model** | χ^2^ | *df* | *p* | RMSEA | CFI | $\Delta$χ^2^ | *df* | *p* |
| ACE: Negative Urgency with Corr Factors  Psychopathology | 994.21 | 311 | < .001 | .077 | .898 |  |  |  |
| Without rA Neg Urgency w/ INT | 994.30 | 312 | < .001 | .076 | .898 | .08 | 1 | .773 |
| Without rA Neg Urgency w/ EXT | 999.15 | 312 | < .001 | .077 | .898 | 4.93 | 1 | .026 |
| Without rA Neg Urgency w/ Att | 1000.08 | 312 | < .001 | .077 | .898 | 5.87 | 1 | .015 |
| Without rA Neg Urgency w/ Soc | 995.81 | 312 | < .001 | .077 | .898 | 1.60 | 1 | .206 |
| Without rA Neg Urgency w/ Thought | 994.21 | 312 | < .001 | .076 | .898 | .00 | 1 | .975 |
| Without rE Neg Urgency w/ INT | 1000.28 | 312 | < .001 | .077 | .898 | 6.07 | 1 | .014 |
| Without rE Neg Urgency w/ EXT | 1002.12 | 312 | < .001 | .077 | .898 | 7.91 | 1 | .005 |
| Without rE Neg Urgency w/ Att | 995.67 | 312 | < .001 | .077 | .898 | 1.46 | 1 | .228 |
| Without rE Neg Urgency w/ Soc | 997.23 | 312 | < .001 | .077 | .898 | 3.01 | 1 | .083 |
| Without rE Neg Urgency w/ Thought | 999.52 | 312 | < .001 | .077 | .898 | 5.30 | 1 | .021 |
| ACE: Lack of Planning with Corr Factors  Psychopathology | 999.43 | 311 | < .001 | .077 | .898 |  |  |  |
| Without rA Planning w/ INT | 1004.79 | 312 | < .001 | .077 | .897 | 5.36 | 1 | .021 |
| Without rA Planning w/ EXT | 1006.84 | 312 | < .001 | .077 | .897 | 7.41 | 1 | .006 |
| Without rA Planning w/ Att | 1007.42 | 312 | < .001 | .077 | .897 | 7.99 | 1 | .005 |
| Without rA Planning w/ Soc | 1003.92 | 312 | < .001 | .077 | .897 | 4.49 | 1 | .034 |
| Without rA Planning w/ Thought | 1006.28 | 312 | < .001 | .077 | .897 | 6.85 | 1 | .009 |
| Without rE Planning w/ INT | 1005.27 | 312 | < .001 | .077 | .897 | 5.84 | 1 | .016 |
| Without rE Planning w/ EXT | 1004.23 | 312 | < .001 | .077 | .897 | 4.80 | 1 | .028 |
| Without rE Planning w/ Att | 999.52 | 312 | < .001 | .077 | .898 | .09 | 1 | .765 |
| Without rE Planning w/ Soc | 999.51 | 312 | < .001 | .077 | .898 | .08 | 1 | .772 |
| Without rE Planning w/ Thought | 999.63 | 312 | < .001 | .077 | .898 | .20 | 1 | .658 |
| ACE: Sensation Seeking with Corr Factors  Psychopathology | 984.02 | 315 | < .001 | .075 | .900 |  |  |  |
| Without rA Sensation Seeking w/ INT | 987.09 | 316 | < .001 | .075 | .900 | 3.07 | 1 | .080 |
| Without rA Sensation Seeking w/ EXT | 984.04 | 316 | < .001 | .075 | .900 | .02 | 1 | .877 |
| Without rA Sensation Seeking w/ Att | 984.83 | 316 | < .001 | .075 | .900 | .81 | 1 | .367 |
| Without rE Sensation Seeking w/ INT | 995.48 | 316 | < .001 | .076 | .898 | 11.46 | 1 | .001 |
| Without rE Sensation Seeking w/ EXT | 987.28 | 316 | < .001 | .075 | .900 | 3.27 | 1 | .071 |
| Without rE Sensation Seeking w/ Att | 987.15 | 316 | < .001 | .075 | .900 | 3.14 | 1 | .077 |
| ACE: Positive Urgency with Corr Factors  Psychopathology | 976.75 | 311 | < .001 | .076 | .900 |  |  |  |
| Without rA Pos Urgency w/ INT | 976.78 | 312 | < .001 | .075 | .901 | .03 | 1 | .869 |
| Without rA Pos Urgency w/ EXT | 982.11 | 312 | < .001 | .076 | .900 | 5.36 | 1 | .021 |
| Without rA Pos Urgency w/ Att | 985.61 | 312 | < .001 | .076 | .899 | 8.86 | 1 | .003 |
| Without rA Pos Urgency w/ Soc | 978.98 | 312 | < .001 | .076 | .900 | 2.23 | 1 | .135 |
| Without rA Pos Urgency w/ Thought | 977.10 | 312 | < .001 | .075 | .901 | .35 | 1 | .556 |
| Without rE Pos Urgency w/ INT | 976.83 | 312 | < .001 | .075 | .901 | .08 | 1 | .780 |
| Without rE Pos Urgency w/ EXT | 976.78 | 312 | < .001 | .075 | .901 | .03 | 1 | .854 |
| Without rE Pos Urgency w/ Att | 976.90 | 312 | < .001 | .075 | .901 | .15 | 1 | .697 |
| Without rE Pos Urgency w/ Soc | 976.76 | 312 | < .001 | .075 | .901 | .01 | 1 | .920 |
| Without rE Pos Urgency w/ Thought | 976.93 | 312 | < .001 | .075 | .901 | .18 | 1 | .674 |
| ACE: Lack of Perseverance with Corr Factors Psychopathology | 988.11 | 311 | < .001 | .076 | .900 |  |  |  |
| Without rA Perseverance w/ INT | 1000.29 | 312 | < .001 | .077 | .898 | 12.18 | 1 | < .001 |
| Without rA Perseverance w/ EXT | 995.44 | 312 | < .001 | .076 | .899 | 7.33 | 1 | .007 |
| Without rA Perseverance w/ Att | 1002.77 | 312 | < .001 | .077 | .898 | 14.66 | 1 | < .001 |
| Without rA Perseverance w/ Soc | 995.26 | 312 | < .001 | .076 | .899 | 7.15 | 1 | .008 |
| Without rA Perseverance w/ Thought | 1000.18 | 312 | < .001 | .077 | .898 | 12.07 | 1 | .001 |
| Without rE Perseverance w/ INT | 995.28 | 312 | < .001 | .076 | .899 | 7.17 | 1 | .007 |
| Without rE Perseverance w/ EXT | 988.15 | 312 | < .001 | .076 | .900 | .04 | 1 | .843 |
| Without rE Perseverance w/ Att | 988.39 | 312 | < .001 | .076 | .900 | .28 | 1 | .595 |
| Without rE Perseverance w/ Soc | 988.78 | 312 | < .001 | .076 | .900 | .67 | 1 | .414 |
| Without rE Perseverance w/ Thought | 991.89 | 312 | < .001 | .076 | .899 | 3.78 | 1 | .052 |

*Note.* A=additive genetic influences, C=shared environmental influences, E=nonshared environmental influences. EXT = Externalizing factor with rule-breaking behavior and aggressive behavior as indicators; INT = Internalizing factor with anxious/depressed, withdrawn/depressed, and somatic complaints as indicators; Att = attention problems; Soc = social problems; Thought = thought problems. Indented models are nested within preceding non-indented models, $\Delta$χ^2^ *p* < .05 indicates a significant reduction in model fit and that a significant parameter was dropped from the model.

**Supplemental Table 17**

*Model Comparisons from Genetic Models of Bifactor Psychopathology Model*

|  | Model Fit | | | | | Model fit vs. full Model | | |
| --- | --- | --- | --- | --- | --- | --- | --- | --- |
| **Model** | χ^2^ | *df* | *p* | RMSEA | CFI | $\Delta$χ^2^ | *df* | *p* |
| ACE: Bifactor Psychopathology | 963.66 | 252 | < .001 | .087 | .893 |  |  |  |
| Without cINT | 963.66 | 253 | < .001 | .087 | .893 | 0.00 | 1 | 1.000 |
| Without cEXT | 963.66 | 253 | < .001 | .087 | .893 | 0.00 | 1 | 1.000 |
| Without cP | 969.41 | 253 | < .001 | .087 | .892 | 5.75 | 1 | .017 |
| Without rA INT w/ EXT | 972.64 | 253 | < .001 | .087 | .891 | 8.98 | 1 | .003 |
| Without aP | 1008.86 | 253 | < .001 | .089 | .886 | 45.20 | 1 | < .001 |
| without rE INT w/ EXT | 991.79 | 253 | < .001 | .088 | .889 | 28.13 | 1 | < .001 |
| Without eP | no convergence | |  |  |  |  |  |  |
| Without aP & cP | 1293.75 | 254 | < .001 | .105 | .843 | 1293.75 | 2 | < .001 |

*Note.* A=additive genetic influences, C=shared environmental influences, E=nonshared environmental influences. EXT = Externalizing factor with rule-breaking behavior and aggressive behavior as indicators; INT = Internalizing factor with anxious/depressed, withdrawn/depressed, and somatic complaints as indicators; P = Common psychopathology factor. Indented models are nested within preceding non-indented models, $\Delta$χ^2^ *p* < .05 indicates a significant reduction in model fit and that a significant parameter was dropped from the model.

**Supplemental Table 18**

*Model Comparisons from Genetic Models of EFs with Bifactor Psychopathology Model*

|  | Model Fit | | | | | Model fit vs. full Model | | |
| --- | --- | --- | --- | --- | --- | --- | --- | --- |
| **Model** | χ^2^ | *df* | *p* | RMSEA | CFI | $\Delta$χ^2^ | *df* | *p* |
| ACE: EFs with Bifactor Psychopathology | 1533.14 | 674 | < .001 | .058 | .884 |  |  |  |
| Without rA EF w/ INT | 1537.24 | 675 | < .001 | .058 | .883 | 4.10 | 1 | .043 |
| Without rA EF w/ EXT | 1533.14 | 675 | < .001 | .058 | .884 | 0.00 | 1 | 1.000 |
| Without rA EF w/ P | 1538.25 | 675 | < .001 | .058 | .883 | 5.11 | 1 | .024 |
| Without rA UPD w/ INT | 1533.17 | 675 | < .001 | .058 | .884 | 0.02 | 1 | .879 |
| Without rA UPD w/ P | 1535.35 | 675 | < .001 | .058 | .884 | 2.21 | 1 | .137 |
| Without rE EF w/ INT | 1533.91 | 675 | < .001 | .058 | .884 | 0.76 | 1 | .382 |
| Without rE EF w/ EXT | 1535.02 | 675 | < .001 | .058 | .884 | 1.87 | 1 | .171 |
| Without rE EF w/ P | 1535.36 | 675 | < .001 | .058 | .884 | 2.21 | 1 | .137 |

*Note.* A=additive genetic influences, C=shared environmental influences, E=nonshared environmental influences. EF = Common EF; UPD = Updating-Specific; EXT = Externalizing factor with rule-breaking behavior and aggressive behavior as indicators; INT = Internalizing factor with anxious/depressed, withdrawn/depressed, and somatic complaints as indicators; P = Common psychopathology factor. Indented models are nested within preceding non-indented models, $\Delta$χ^2^ *p* < .05 indicates a significant reduction in model fit and that a significant parameter was dropped from the model.

**Supplemental Table 19**

*Model Comparisons from Genetic Models of Impulsivity Facets with Bifactor Psychopathology Model*

|  | Model Fit | | | | | Model fit vs. full Model | | |
| --- | --- | --- | --- | --- | --- | --- | --- | --- |
| **Model** | χ^2^ | *df* | *p* | RMSEA | CFI | $\Delta$χ^2^ | *df* | *p* |
| ACE: Negative Urgency with Bifactor Psychopathology | 1050.11 | 323 | < .001 | .078 | .892 |  |  |  |
| Without rA Neg Urgency w/ INT | 1050.873 | 324 | < .001 | .077 | .892 | 0.76 | 1 | .383 |
| Without rA Neg Urgency w/ EXT | 1051.348 | 324 | < .001 | .077 | .892 | 1.24 | 1 | .266 |
| Without rA Neg Urgency w/ P | 1051.672 | 324 | < .001 | .077 | .892 | 1.56 | 1 | .212 |
| Without rE Neg Urgency w/ INT | 1050.65 | 324 | < .001 | .077 | .892 | 0.54 | 1 | .462 |
| Without rE Neg Urgency w/ EXT | 1051.02 | 324 | < .001 | .077 | .892 | 0.91 | 1 | .340 |
| Without rE Neg Urgency w/ P | 1055.87 | 324 | < .001 | .078 | .891 | 5.76 | 1 | .016 |
| ACE: Lack of Planning with Bifactor Psychopathology | 1056.17 | 323 | < .001 | .078 | .891 |  |  |  |
| Without rA Planning w/ INT | 1056.21 | 324 | < .001 | .078 | .891 | 0.05 | 1 | .832 |
| Without rA Planning w/ EXT | 1056.24 | 324 | < .001 | .078 | .891 | 0.07 | 1 | .786 |
| Without rA Planning w/ P | 1062.69 | 324 | < .001 | .078 | .890 | 6.53 | 1 | .011 |
| Without rE Planning w/ INT | 1063.41 | 324 | < .001 | .078 | .890 | 7.25 | 1 | .007 |
| Without rE Planning w/ EXT | 1060.41 | 324 | < .001 | .078 | .890 | 4.24 | 1 | .039 |
| Without rE Planning w/ P | 1056.45 | 324 | < .001 | .078 | .891 | 0.28 | 1 | .597 |
| ACE: Sensation Seeking with Bifactor Psychopathology | 1033.70 | 325 | < .001 | .076 | .894 |  |  |  |
| Without rA Sensation Seeking w/ INT | 1036.63 | 326 | < .001 | .076 | .894 | 2.93 | 1 | .087 |
| Without rA Sensation Seeking w/ EXT | 1033.78 | 326 | < .001 | .076 | .894 | 0.08 | 1 | .777 |
| Without rE Sensation Seeking w/ INT | 1047.11 | 326 | < .001 | .077 | .892 | 13.41 | 1 | < .001 |
| Without rE Sensation Seeking w/ EXT | 1037.07 | 326 | < .001 | .076 | .894 | 3.37 | 1 | .066 |
| ACE: Positive Urgency with Bifactor Psychopathology | 1035.54 | 322 | < .001 | .077 | .893 |  |  |  |
| Without rA Pos Urgency w/ INT | 1039.57 | 323 | < .001 | .077 | .893 | 4.03 | 1 | .045 |
| Without rA Pos Urgency w/ EXT | 1036.06 | 323 | < .001 | .077 | .893 | 0.52 | 1 | .473 |
| Without rA Pos Urgency w/ P | 1035.56 | 323 | < .001 | .077 | .893 | 0.02 | 1 | .896 |
| Without rE Pos Urgency w/ INT | 1035.92 | 323 | < .001 | .077 | .893 | 0.38 | 1 | .535 |
| Without rE Pos Urgency w/ EXT | 1035.54 | 323 | < .001 | .077 | .893 | 0.00 | 1 | .975 |
| Without rE Pos Urgency w/ P | 1035.75 | 323 | < .001 | .077 | .893 | 0.21 | 1 | .644 |
| ACE: Lack of Perseverance with Bifactor Psychopathology | 1055.61 | 323 | < .001 | .078 | .892 |  |  |  |
| Without rA Perseverance w/ INT | 1055.62 | 324 | < .001 | .078 | .892 | 0.01 | 1 | .938 |
| Without rA Perseverance w/ EXT | 1056.39 | 324 | < .001 | .078 | .892 | 0.78 | 1 | .378 |
| Without rA Perseverance w/ P | 1068.36 | 324 | < .001 | .078 | .890 | 12.75 | 1 | < .001 |
| Without rE Perseverance w/ INT | 1059.40 | 324 | < .001 | .078 | .891 | 3.79 | 1 | .051 |
| Without rE Perseverance w/ EXT | 1056.28 | 324 | < .001 | .078 | .892 | 0.67 | 1 | .413 |
| Without rE Perseverance w/ P | 1056.17 | 324 | < .001 | .078 | .892 | 0.57 | 1 | .452 |

*Note.* A=additive genetic influences, C=shared environmental influences, E=nonshared environmental influences. EXT = Externalizing factor with rule-breaking behavior and aggressive behavior as indicators; INT = Internalizing factor with anxious/depressed, withdrawn/depressed, and somatic complaints as indicators; P = Common psychopathology factor. Indented models are nested within preceding non-indented models, $\Delta$χ^2^ *p* < .05 indicates a significant reduction in model fit and that a significant parameter was dropped from the model.
